# Slot-Die Coated Triple-Halide Perovskites for Efficient and Scalable Perovskite/Silicon Tandem Solar Cells

**DOI:** 10.1021/acsenergylett.2c01506

**Published:** 2022-09-27

**Authors:** Ke Xu, Amran Al-Ashouri, Zih-Wei Peng, Eike Köhnen, Hannes Hempel, Fatima Akhundova, Jose A. Marquez, Philipp Tockhorn, Oleksandra Shargaieva, Florian Ruske, Jiahuan Zhang, Janardan Dagar, Bernd Stannowski, Thomas Unold, Daniel Abou-Ras, Eva Unger, Lars Korte, Steve Albrecht

**Affiliations:** † Department Perovskite Tandem Solar Cells, Helmholtz-Zentrum Berlin für Materialien und Energie GmbH, Kekuléstraße 5, 12489 Berlin, Germany; ‡ Competence Centre Photovoltaics (PVcomB), Helmholtz-Zentrum Berlin, Schwarzschildstraße 3, 12489 Berlin, Germany; § Department of Structure and Dynamics of Energy Materials, Helmholtz-Zentrum Berlin für Materialien und Energie GmbH, 14109 Berlin, Germany; ∥ Department Solution-Processing of Hybrid Materials and Devices, Helmholtz-Zentrum Berlin für Materialien und Energie GmbH, Kekuléstraße 5, 12489 Berlin, Germany; ⊥ Department Novel Materials and Interfaces for Photovoltaic Solar Cells, Helmholtz-Zentrum Berlin für Materialien und Energie GmbH, Kekuléstraße 5, 12489 Berlin, Germany; # Faculty of Electrical Engineering and Computer Science, Technical University Berlin, 10587 Berlin, Germany

## Abstract

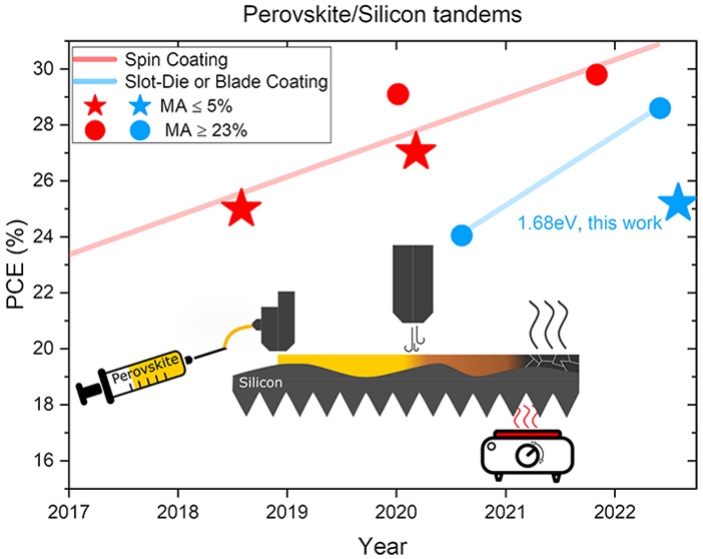

Wide bandgap halide perovskite materials show promising potential to pair with silicon bottom cells. To date, most efficient wide bandgap perovskites layers are fabricated by spin-coating, which is difficult to scale up. Here, we report on slot-die coating for an efficient, 1.68 eV wide bandgap triple-halide (3halide) perovskite absorber, (Cs_0.22_FA_0.78_)Pb(I_0.85_Br_0.15_)_3_ + 5 mol % MAPbCl_3_. A suitable solvent system is designed specifically for the slot-die coating technique. We demonstrate that our fabrication route is suitable for tandem solar cells without phase segregation. The slot-die coated wet halide perovskite is dried by a “nitrogen (N_2_)-knife” with high reproducibility and avoiding antisolvents. We explore varying annealing conditions and identify parameters allowing crystallization of the perovskite film into large grains reducing charge collection losses and enabling higher current density. At 150 °C, an optimized trade-off between crystallization and the PbI_2_ aggregates on the film’s top surface is found. Thus, we improve the cell stability and performance of both single-junction cells and tandems. Combining the 3halide top cells with a 120 μm thin saw damage etched commercial Czochralski industrial wafer, a 2-terminal monolithic tandem solar cell with a PCE of 25.2% on a 1 cm^2^ active area is demonstrated with fully scalable processes.

Tandem solar cells, combining a tunable wide bandgap halide perovskites top cell (around 1.68 eV) with a silicon bottom cell, surpassed the Auger recombination limited single-junction silicon solar cells’ efficiency^1,2^ and have recently reached a world record power conversion efficiency (PCE) of 29.8%.^3^ Both the state-of-the-art halide perovskites single-junction solar cell (25.7% PCE on 0.1 cm^2^)^4^ and the perovskite/silicon tandem solar cell (29.8% PCE on 1 cm^2^)^3^ are manufactured by spin coated perovskite layers. However, for spin-coating, the homogeneity of the thin film on large scale is always a main challenge.^5^ When the active area increases further, it becomes more difficult to control the film thickness from the substrate center toward the edges. In addition, 90% of the halide perovskites precursor and antisolvents are spun off and wasted during the spin-coating process, which is not economically and environmentally viable for mass production.^6^ In addition, antisolvents deposited during spin-coating are typically used to achieve high-quality thin films: they interact with halide perovskites precursors to trigger crystallization of halide perovskites out of the wet film. However, the film formation is still not fully understood and is difficult to control for larger areas. Besides achieving high PCEs close to 30%, the major challenge for perovskite/silicon tandems thus remains to transfer these high PCEs to commercial, large area, mass-produced silicon bottom cells. These have thicknesses ≤ 140 μm and a rough surface (mean arithmetic height, Sa ≈ 0.7 μm) due to the saw damage etching (SDE) process. Spin-coating halide perovskites on such wafers is difficult. The thick perovskite film by slot-die coating (SDC) and insufficient thin wafer absorption need to be further investigated for the optimal current matching condition of the tandem solar cells.

There are several printing techniques available for upscaling, such as blade coating, inkjet printing, and slot-die coating. The blade coating technique is limited by the automation process and ink supply, while inkjet printing is limited by the slow deposition speed and potential nozzle clogging. Equipped with an automatic coating table and continuous ink supply syringe with 100 μm coating slits for the slot-die head, the slot-die coater platform has advantages such as easy operation, easy cleaning, and accurate thickness. To control the drying process of the coated wet film, N_2_ gas flows from so-called N_2_-knives have been widely used to induce film formation^7−9^ similar to the antisolvent drip in spin-coating. Therefore, slot-die coating with N_2_ gas quenching, which is also possible to be upscaled for roll-to-roll production, is a promising candidate for scalable deposition and low cost.^7,10,11^ Another important aspect is to find a wide bandgap halide perovskites ink composition that leads to high crystal quality, no phase segregation, high thermal stability, and high PCE for these coating processes. The state-of-the-art halide perovskites solar cells have been summarized in Figure S1 and Table S1 for the coating techniques of spin-coating and printing in general. Two composition groups have been widely developed for the perovskite/silicon tandem applications, namely triple-cation perovskites comprising cesium (Cs^+^), methylammonium (MA^+^), and formamidinium (FA^+^) as the A-cations^12,13^ and double-cation perovskites containing Cs^+^ and FA^+^.^14,15^ The MA^+^ free perovskites have the advantages of long-term and thermal stability while the performance is a shortcoming due to the limited photovoltage of these halide perovskites materials.^16,17^ According to optical simulations pairing halide perovskites with silicon in a tandem layer stack, the optimal perovskite bandgap is around 1.70 eV;^18^ thus, groups working on tandems have increased the bandgap from 1.63 eV toward 1.68 eV in past efforts. Cs^+^ and Br^–^ are widely utilized to increase the bandgap, but cation and halide segregation have been the main limitation with maximum amounts of 25% Cs and 20% Br reaching 1.68 eV.^19^

Finally, considering the solvent system, the combination of DMF and DMSO has been the most popular system for most of the published compositions for spin-coating due to the high solubility.^20^ However, without the usage of antisolvents, DMSO has been detrimental in scaling to large area deposition assisted by N_2_ quenching. There are also two works reported for tandem solar cells fabricated by printing techniques.^8,9^ However, the bandgap, phase stability, and high MA concentration have been the bottlenecks that limit further development. Although 2-ME (2-mercaptoethanol) has been widely used for slot-die coating,^21,22^ the low solubility of Cs^+^ and Br^–^ hinders their addition to precursor inks and, hence, the bandgap tunability. So far, ink systems with low methylammonium^+^ (MA^+^) content and wide bandgap compositions for printing methods are rarely reported but are essential for the upscaling purpose of perovskite-based tandem solar cells.

Here we present a suitable ink system and film drying process for improved perovskite film quality with high photoluminescence quantum yield when using a so-called triple-halide perovskite with top cell optimized bandgaps.^17^ Additionally, we have integrated the halide perovskites into a tandem architecture with industrial silicon bottom cells, demonstrating scalable industrially relevant perovskite silicon tandem solar cells. The triple-halide perovskites contains chloride (Cl^–^) to reduce the amount of bromide (Br^–^) needed for top cell optimized bandgaps,^17^ also to improve the surface morphology and to induce a surface passivation.^23^ We targeted to include 5 mol % additional MAPbCl_3_ into the well-investigated double-cation perovskites Cs_0.22_FA_0.78_Pb(I_0.85_Br_0.15_)_3_ (1.63 eV) to achieve a bandgap of 1.68 eV with no phase segregation and excellent optoelectronic properties. By optimizing the drying and annealing conditions from 100 to 170 °C for 20 min, the absolute as well as transient photoluminescence (PL) shows obvious improvements in quasi-Fermi level splitting (QFLS) and charge carrier lifetimes. In addition, the film morphology as a function of annealing conditions and the optimized crystallization enable large grain size reducing charge collection losses and, thus, achieving higher current density in solar cells. With annealing at 150 °C, an optimized trade-off between sample crystallinity and the detrimental formation of large PbI_2_ aggregates on the film’s top surface is found. Thus, supreme stability and performance of slot-die coated perovskite single-junction cells toward a stabilized power output of 19.4% are achieved, which is the highest yet reported for halide perovskites with top cell optimized bandgaps. Finally, by integrating the optimized perovskite absorber layers’ fabrication with silicon bottom cells made of commercial Czochralski (Cz) thin wafers (around 120 μm) and no extra chemical or mechanical surface polishing, a two-terminal monolithic tandem solar cell with a PCE of 25.2% on a 1 cm^2^ active area is demonstrated with fully scalable processes. Our findings open the way for efficient perovskite/silicon tandem solar cells with optimized bandgap and scalable fabrication routes via slot-die coating.

## Ink and Film Properties

We start with an investigation of halide perovskites’ film formation and material properties for slot-die coating, so-called “’triple-halide”’ perovskites with N_2_ gas quenching, aiming at investigating with electrical and optical measurements on how the drying method and annealing temperature influence the formation of perovskite films. A spin coated triple-halide (3halide) perovskite with antisolvent treatment is used as reference for comparisons because of its excellent performance.^17^ The used 3halide perovskite precursor composition is Cs_0.22_FA_0.78_Pb(I_0.85_Br_0.15_)_3_ (denoted as Cs22Br15) + 5 mol % MAPbCl_3_. The detailed coating description can be found in the Supporting Information and in Figure S3. A direct comparison of the PL intensity and peak position stability between spin coated (annealed at 100 °C) and slot-die coated (annealed at 150 °C) films on ITO coated glass covered with the self-assembled monolayer (SAM) molecules 2PACz^24^ ([2-(9H-carbazol-9-yl)ethyl]phosphonic acid) can be found in Figure S2 as a function of time. The spin coated double-cation host composition Cs22Br15 has also been utilized as reference. Under 1 sun illumination intensity, all three samples show no obvious peak shift. However, under 10 suns illumination, although no secondary peak is observed, an observable red shift of the PL emission is detected for both deposition methods (possibly enhanced by the small 0.4 mm^2^ spot^12^). We found that, for both spin coated and slot-die coated samples, a slight red shift of the PL is observed indicating some light-induced phase-segregation. However, this perovskite composition is still desirable to be investigated for the solar cell under 1 sun illumination. In addition to the photostability, the low MA^+^ concentration used here for the 3halide perovskite can also prevent degradation under high-temperature treatments, which might be necessary for, for example, screen-printed silver electrodes’ curing formation in future upscaling.^25^ To transfer the recipe to slot-die coating, a new solvent system has been developed in this study: the DMF (dimethylformamide)-based solvent both has high solubility of precursors for the Cs^+^, FA^+^-based double-cation perovskites and is without precipitation during the coating process. In order to stabilize the intermediate stage between the gas quenching and post annealing, *N*-methyl-2-pyrrolidone (NMP) has been utilized to maintain the large intermediate wet film time scale with a wide processing time window between the gas quenching stage and the annealing stage in our work.^26^

**Figure 1:**
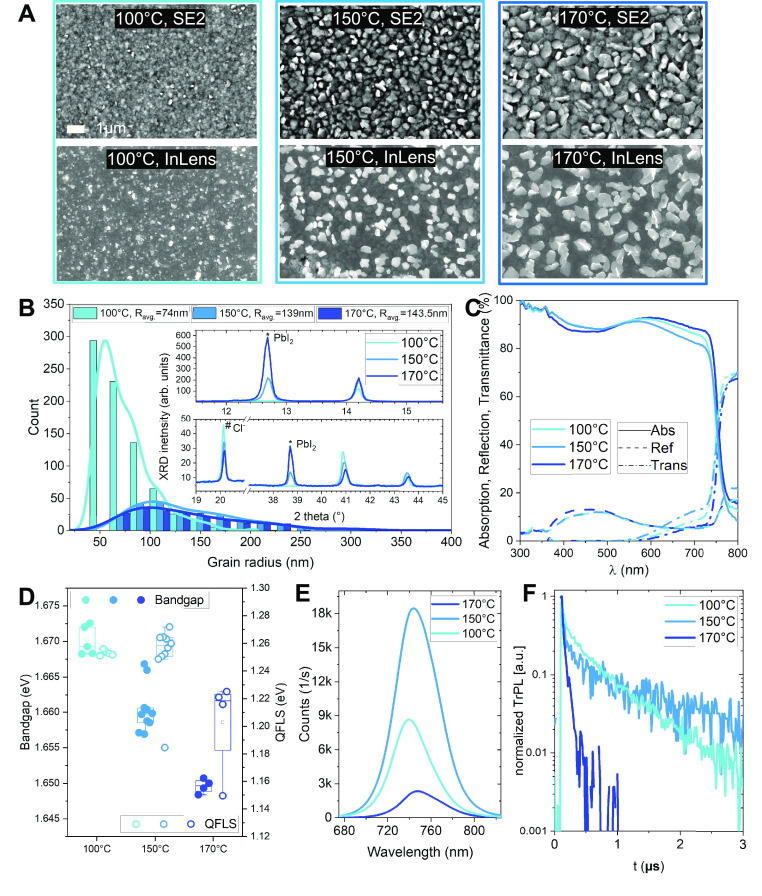
Characterizations of slot-die coated perovskite films on indium tin oxide (ITO) coated glass substrates covered with hole transporting materials (here self-assembled monolayer molecule “’2PACz’”) prepared at various annealing temperatures as indicated. (A) Top-view SEM images showing the perovskite domain size and surface composition distribution; the scale bar in part A applies to all SEM images. The first row is acquired by the secondary electron detector (SE2) showing the halide perovskites topography with high lateral resolution. The second row was recorded using the InLens detector (SE1) which enhances the surface compositional contrast. (B) Grain radius distribution as derived from the first row in panel A. The mean area equalized grain radius (R_avg_) indicates an increased grain size for higher annealing temperatures. Inset: Angular X-ray diffraction (XRD) patterns, rescaled to allow for better comparison of peak positions. (C) UV–vis spectra to show the shift of the absorption edge with annealing temperatures. (D) Photoluminescence (PL) measurement statistics show that samples annealed at 150 °C have the highest calculated QFLS. (E) Corresponding PL spectra. (F) Time-resolved photoluminescence (trPL) transients, showing the slowest nonradiative recombination for a film annealed at 150 °C.

Here we will specifically focus on the properties of annealed halide perovskites films. After slot-die coating of the precursor solutions on substrates covered with indium tin oxide (ITO) and hole selective SAMs from our previous studies,^24^ the printed and N_2_-knife dried perovskite films were annealed at different temperatures (100, 150, and 170 °C) for 20 min, aiming for optimized film formation. Occasionally, we observed that the slot-die coated films annealed at 100 °C turn yellowish after exposure for 12 h to ambient air while the film annealed at 150 °C kept the black color for at least 5 days in 60% relative humidity ambient air. At the same time, for the antisolvent treated spin coated perovskite film which has been annealed at 100 °C, the color remains black in the same ambient condition. The different vulnerability to moisture and oxygen is a first indication that a different perovskite composition and morphology have been formed. Meanwhile, the remaining solvate phases could also be formed due to the remaining solvents by the gas quenching process. In previous reports, numerous studies and investigations about the phase instability of FAPbI_3_ were reported.^27,28^ Several methods were developed to keep the FAPbI_3_ in the α-phase, such as increasing the annealing temperature or the incorporation of Cs and additives such as methylammonium chloride (MACl) and formamidinium chloride (FACl) into the precursor solution.^29,30^ However, all investigations were carried out by spin-coating with antisolvents treatment. Thus, we hypothesized that the crystallization and wanted perovskite phase formation could also proceed differently under N_2_ quenching conditions. Therefore, we increased the annealing temperature further toward 170 °C, which was the boundary temperature to enter the α-FAPbI_3_ crystal structure,^27^ and investigated the film’s surface morphology by using scanning electron microscopy (SEM) with both the secondary electron detector (SE2) and InLens detector (SE1), which are more sensitive to surface height topography and to surface composition reflected by the gray contrast, respectively. The apparent “’grain’” sizes of the SEM images in the top row of Figure 1A were analyzed with a modified algorithm applied to generate the equivalent grain radius distribution (see Experimental Procedures in the Supporting Information for further information) in Figure 1B.^31^ The algorithm calculates the size of the grain area, and then the same size circular area represents the calculated grain area. At last, the circular radius can be summarized in a histogram for easiness of comparison. This analysis shows clearly that higher annealing temperatures significantly enlarge the domain size from an average of 74 nm for 100 °C annealing to 139 nm for 150 °C and finally to 144 nm for 170 °C. In the next step, we examined the crystal properties by XRD, as shown in the inset of Figure 1B. A detailed comparison between the 100 °C annealed spin coated reference sample and the 150 °C annealed slot-die coated sample is also listed in Figure S4. The characteristic halide perovskites 2θ peak (14.1°, 100) intensity doubled when the temperature increased from 100 to 150 °C, which implies the enhanced crystallinity.^32^ The XRD pattern showed no further change at 14.1° when the temperature increased from 150 to 170 °C. The peaks at 12.7° and 38.7° correspond to the (001) PbI_2_ diffraction plane, and the 20.1° and 40.8° peaks indicate the existence of Cl^–^ species (110) in the lattice.^17^ This proves the decreased inclusion of Cl^–^ within the halide perovskites lattice for slot-die coating, while secondary PbI_2_ phases are generated during the 150 and 170 °C annealing steps. Meanwhile the Br-rich grains are also observed by energy-dispersive X-ray (EDX) in Figure S5, which is caused by the fast crystallization process from solution.^33^ The lower amount of Cl^–^ is expected to reduce the bandgap, which is confirmed in Figure 1D and E.^17^ We speculate that the higher thermal budget (170 °C) induces a lower methylammonium chloride (MACl) concentration during the 20 min annealing process. The PbI_2_ is clear by the XRD peaks at 12.7° (2.7-fold increase) and 38.7° (2.3-fold increase) in Figure 1B. The peak intensity starts to rise rapidly when the temperature is higher than 150 °C. The slot-die coated 3halide film shows no phase segregation that would be visible in the XRD pattern in the range between 15° and 15.5°, a finding that has been discussed in previous reports.^17^

In comparison with the slot-die coated 3halide film, the spin coated 3halide film shows no extra PbI_2_ signal under XRD inspection.^17^ The degassing from the surface and bulk might lead to a gradient formation expected with higher annealing temperatures. It was important to find out whether the PbI_2_ excess is located at the surface or in the bulk. Hence, a cross-sectional EDX was measured for samples annealed at 100, 150, and 170 °C (Figures S7–S9). Apart from an increased bromide concentration toward the surface, there is no direct evidence of an uneven gradient between I^–^ and Pb^2+^ from the cross-sectional EDX images. Thus, from these measurements there is no indication that the halide perovskites have decomposed into PbI_2_ with high volume fraction. Furthermore, grazing incidence XRD (GIXRD) is utilized to investigate the perovskite surface with high sensitivity (see Figure S10) by comparing the intensity ratio of the diffraction peaks at 12.7° (PbI_2_):14.1° (halide perovskites). For higher incidence angles, the measurements become more sensitive to the perovskite bulk film crystallographic information. At an incidence angle of 3°, the intensity ratio decreases for all samples, and the sample annealed at 170 °C still gave the highest value. This suggests that the secondary phase of PbI_2_ mainly is situated on the perovskite thin film top surface and that the relative amount increases with annealing temperature. This observation is consistent with the increased amount of flake-like features with higher brightness observed for samples annealed at higher temperatures in SEM images.

The absorption spectrum (UV–vis) shows the optical property of the slot-die coated films (Figure 1C). With a 600 nm slot-die coated perovskites thin film, the absorption of more than 80% of the incident light between the wavelengths of 300 and 740 nm proves that these films can convert photons into charge carriers effectively. To measure the bandgap and estimate the quasi-Fermi level splitting (QFLS) of the perovskite (see Experimental Procedures in the Supporting Information), we utilize the absolute photoluminescence of the halide perovskites deposited on glass/ITO/SAM(2PACz) layer stacks as shown in Figure 1D. The bandgap decreased linearly with the temperature increment from 1.68 eV for 100 °C to 1.66 eV for 170 °C annealing. Despite the intermediate bandgap at 1.67 eV with 150 °C annealed samples, the QFLS shows the highest value among the annealing conditions at 1.26 eV, indicating low nonradiative recombination for 150 °C annealing. The spin coated 3halide reference sample (annealed at 100 °C) reaches 1.27 eV. The Gaussian shape fit of the slightly asymmetrical PL spectral result indicates a slightly lower bandgap compared with the bandgap derived from the external quantum efficiency (EQE) later.^34^ The PL spectra shown in Figure 1E are also another direct evidence for the bandgap shift, as seen from the peak emission.

Time-resolved photoluminescence (trPL) of halide perovskites films was measured for the three annealing conditions on ITO coated glass with 2PACz as the hole transport layer and is displayed in Figure 1F. The fast initial decay can be attributed to fast hole extraction into the ITO.^35^ The more slowly decaying component at longer times is dominated by nonradiative charge carrier recombination through defects,^36^ which is slowest for the 150 °C annealing condition. Furthermore, the trend in carrier lifetimes for different annealing temperatures behavior also correlates well with the discussed QFLS and PLQY (photoluminescence quantum yield) in Figure 1D and E. This further proves the reduced nonradiative recombination for 150 °C annealed samples. However, the 170 °C annealed sample shows the fastest nonradiative recombination although it exhibits the largest domain size, which contradicts our expectation of recombination happening mostly at the grain boundaries.^37^ To find out the reasons behind the misalignment of the grain size and PL lifetime for 170 °C annealed samples, energy-dispersive X-ray (EDX) analysis was applied to examine the compositional distribution from both cross-sectional and top-view images. The elemental images in Figure S5 reveal that Br atoms were the only chemical element that was not evenly distributed in the film. Further, we collected the averaged compositional intensity across the top surface over a large area for each composition. From this, it is apparent that the chlorine content decreases with increasing annealing temperature (see also Table S2). In contrast, the lead (Pb) concentration increases with temperature. For iodine (I), the same trend can also be observed. The EDX elemental intensity distribution (Figure S6) is composed from two parts when we investigate the whole energy scanning from 0 eV to 4 keV: low energy 0.4 keV (280 nm penetration depth), which carries a larger portion of information for the surface, and 3.9 keV (3720 nm penetration depth), which can reflect the bulk property within a large volume. There is no clear difference for the 3.9 keV scanning region, which hints at similar bulk properties. The intensity of the 0.4 keV scanning region gives an observable difference and implies a surficial I^–^ concentration difference. Thus, we conclude that the excess I^–^ is mainly found on the halide perovskites’ top surface.

## Highly Efficient Wide Bandgap Halide Perovskite Single-Junction Solar Cells

**Figure 2:**
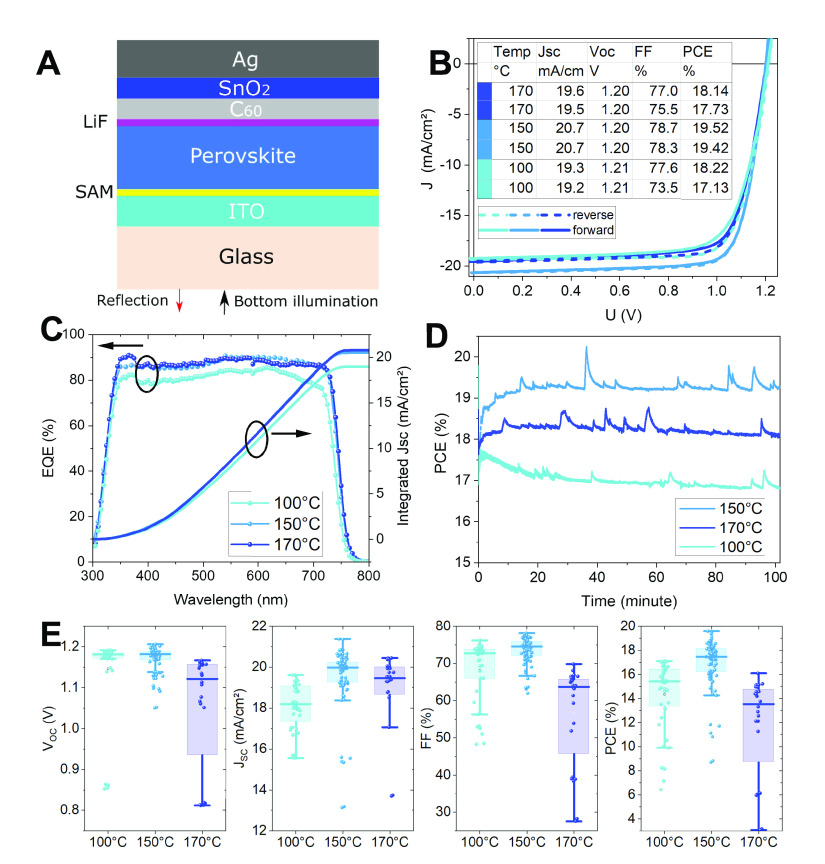
Solar cell characteristics of single-junctions for annealing temperatures of 100, 150, and 170 °C. (A) Schematic of the single-junction device with illumination through the glass and an active area of 0.16 cm^2^. (B) *J*–*V* curves of the best solar cells for each annealing temperature. Solid lines: forward scan (from *V*_oc_ to *J*_sc_); dashed lines: reverse scan. Inset table: corresponding device parameters. (C) External quantum efficiency spectra (EQE, left axis) and integrated short circuit current density (right axis) for the cells in panel B. (D) Continuous maximum-power-point (MPP) track of the best solar cells for 100 min at 25 °C. (E) Box plot graphs of characteristic solar cell device parameters for investigated annealing temperatures. The data set contains 74 solar cells in total.

To investigate the solar cell performance, we first compare slot-die coated single-junction solar cells with the following layer stack: either BCP (bathocuproine) or SnO_2_ between the fullerene (C_60_) and Ag (or Cu) contacts. The former is typically used for opaque p-i-n solar cells, while the latter is needed for top contacts in tandem solar cells. There are no observable performance differences. Thus, we utilize SnO_2_ in single-junctions that can later serve as a buffer layer for the sputtered indium zinc oxide (IZO) electrode required for tandem application. Figure 2A shows the corresponding device configuration, and Figure 2B shows the best *J*–*V* data under the investigated annealing conditions. In combination with Figure 2D (showing the maximum power point tracking) and Figure 2E (showing statistical data of the performance parameters for 74 solar cells), they reveal the overall trend and explain how the annealing temperature impacts on the full solar cell performance. The current density of the sample annealed at 100 °C shows only 18 mA/cm^2^ on average. Although the absorption coefficients are approximately in a similar level among all annealing temperatures shown in Figure 1C, the poor charge collection from the low EQE signal for 100 °C annealed films in the complete wavelength between 350 and 700 nm proves the high recombination caused by the small grain size and, hence, large limitation, presumably due to recombination in grain boundaries.^32^ In Figure S15, the 11 h maximum-power-point (MPP) track also shows the instability caused by the small grain size with dramatically decreased current density. Hence, annealing at 100 °C, which has been commonly used for spin-coating, cannot be easily transferred to trigger correct crystallization under SDC and N_2_-knife drying conditions. In Figure 2C, the calculated EQE derivative from 150 °C shows the bandgap at 1.68 eV, which is desirable to pair with the silicon solar cell. The 150 °C annealed sample shows the highest *V*_oc_, fill factor (FF), and *J*_sc_ and, thus, the highest efficiency for all temperatures investigated here: the best 150 °C sample stabilized at 19.4% efficiency, with *V*_oc_ and *J*_sc_ values of 1.2 V and 20.65 mA/cm^2^, which are the highest reported for slot-die coated 1.68 eV top cell optimized perovskite solar cells. In Figure 2D, the MPP tracks measured for 100 min in inert atmosphere are shown. These stability tests also demonstrate that 150 °C is the optimal temperature for annealing with no difference between the *J*–*V* scan and the MPP track. The spikes in the MPP track are caused by sudden changes of the glovebox pressure during operation and do not reflect a sample characteristic. The 300 h shelf-lifetime also demonstrates the good stability in Figure S16. When the annealing temperature increases from 150 °C further to 170 °C, the *V*_oc_ and FF decrease significantly. The *J*–*V* curve in Figure S12 demonstrates that this is caused by higher series resistance. The accumulated PbI_2_ platelets might provide high resistance and strong recombination pathways, as we have seen from the absolute and transient PL above. It is conceivable that, when the 170 °C samples undergo two subsequent *J*–*V* scans with a waiting time of 2 min light soaking, the ions accumulate and build up between the deteriorated perovskite/C_60_ interface as an additional internal field which hinders the carrier extraction and, subsequently, the *J*_sc_ is reduced, as seen in Figure 2B.^38^ The XRD measurement which has been discussed previously can give a deeper interpretation. Although the general Pb^2+^ and I^–^ distribution is kept even in the bulk layer (Figure S7), the surface-near PbI_2_ platelets are the most probable reason for the higher series resistance although there is no excess PbI_2_ involved in the preparation stage in our fabrication. Meanwhile, the *V*_oc_ reduction is in line with the 40 mV reduction of the QFLS which we have discussed in the last section, although the bandgap only decreases by 10 mV in the comparison between the 100 and 150 °C annealing conditions.

The best slot-die coated single-junction solar cells developed here with annealing at 150 °C reach FF values around 78%, while spin coated halide perovskites of the same composition can reach FFs above 81%.^17^ To better understand the limited FFs of slot-die coated solar cells, we prepared three kinds of samples on the quartz glass: spin coated 3cation^12^ perovskites as reference, spin coated 3halide perovskites (3halide SC), and slot-die coated 3halide perovskites (3halide SDC). The aims are to compare both the coating methods and two recipes to evaluate the halide perovskites quality, interface-near and bulk defects, and its impact on PL behavior. We measured trPL and found that the spin coated 3halide film shows the longest carrier lifetime of close to 3 μs, as seen in Figure S13. Although the spin coated 3halide shows smaller grain size than the slot die coated 3halide, as seen in Figure S14, photogenerated carriers in the slot-die coated 3halide film decay with a lifetime of only 0.7 μs. This obvious difference in PL lifetime between spin and slot die coated 3halide films can be interpreted by the limited bulk quality and can explain the lower FF for slot-die coated samples.

From another viewpoint, the diode ideality factor (n_id_) and potential fill factor (pFF) are widely used to evaluate the dominant recombination pathways in semiconductors.^39,40^ We further analyzed various films from spin-coating and slot-die coating on ITO and ITO/silicon substrates with intensity-dependent PL to extract pseudo fill factors.^39^ In Table 1, data for these metrics are collected from intensity-dependent PL measurement, according to fits shown in Figure S11. Comparing spin-coating and slot-die coating (Table 1, a vs b), the halide perovskites film shows almost no difference in ideality factor, with values below 1.3 indicating a dominance of Shockley–Read–Hall recombination through traps and defects within the band gap, while the PLQY of SC halide perovskites is still higher than the SDC one which leads to the higher p*V*_oc_. Comparing the coating techniques between SC and SDC, the existence of PbI_2_ suggests a potentially stronger nonradiative recombination with lower photoluminescence property. When we compare the ΔFF values, which are derived by the pFF and real solar cell *J*–*V*, it can clearly illustrate the different halide perovskites films induced by the spin-coating and slot-die coating methods. The ΔFF values reflect that the series resistance is still higher for the slot-die coated film, which aligns with our previous discussion. Further investigation is still necessary, and this also highlights that those potential halide perovskites film improvements need to be studied with the slot-die coating process presented here for further optimized crystallization. When the substrates have been compared between ITO glass and ITO coated silicon substrates (Table 1, b vs c), no obvious difference exists for the perovskite film apart from the lower photoluminescent emission. In the PL setup, the illumination occurs on the perovskite side of the sample. A white reflectance holder for substrates is utilized as the same function of the integrate sphere to reflect the PL signal from the ITO glass or silicon substrates. Therefore, the lower PL signal on the substrate is likely caused by the reabsorption of the silicon bottom cell.^41^

**Table 1:** Ideality Factor (n_id_) and Pseudo Fill Factor (pFF) for Halide Perovskite Films on Various Substrates Calculated from Intensity-Dependent PL Measurements^a^

Sample	n_id_	J_EQE_ (mA/cm^2^)	p*V*_oc_ (V)	pFF (%)	pPCE (%)	*V*_oc_ (V)	FF (%)	ΔFF = pFF – FF, Δ*V*_oc_ = p*V*_oc_ – *V*_oc_
a. SC on 2PACz/ITO	1.29	20.5	1.29	88.2	23.3	1.25	81.0	7.2, 0.04
b. SDC on 2PACz/ITO	1.27	20.5	1.26	87.2	22.7	1.22	78.0	9.2, 0.04
c. SDC on 2PACz/ITO/silicon	1.26	20.5	1.25	87.5	22.5			

a The halide perovskites were coated on quartz glass and ITO coated glass by spin-coating or slot-die coating. The measurement data and the curve fits are displayed in Figure S11. In addition, the best fill factor (FF) from solar cell devices and the difference between the pseudo and solar cell fill factors (ΔFF) and pseudo *V*_oc_ vs *V*_oc_ (Δ*V*_oc_) are presented.

Finally, 150 °C annealing is the optimal condition for SDC 3halide perovskites, and it will be further utilized in the tandem solar cell section.

## Integration of Slot-Die Coated Triple-Halide Perovskites into Silicon-Based Tandem Solar Cells

We now turn to the integration of slot-die coated perovskites top cells on silicon bottom cells for two-terminal (2T) monolithic tandem solar cell integration. During slot-die coating of silicon cells, it is unavoidable that the perovskite precursor solution flows around the wafer’s edge and reaches its back side, leading to destruction, indicated by discoloration of the Ag rear contact and decreased cell performance. Therefore, to improve the reproducibility of slot-die coated tandem solar cells, we investigated the reaction between the halide perovskites solution and the back contact materials in detail. The results are presented from Figure S19 to Figure S21 in the Supporting Information. Briefly, it appears that the lead halide solution reacts with the Ag electrode to form silver halides,^42^ which are nonconductive and difficult to dissolve. To avoid these reactions and improve reproducibility, 25 nm SiO_*x*_ has been added on the back side of the silicon wafer before slot-die coating to protect the back silver electrode. After the whole fabrication process, 1% HF solution dipping has been utilized to remove the SiO_*x*_ protection layer. With this, we could utilize the wide bandgap 3halide perovskites in tandem solar cells and investigate the losses in comparison with the standard spin coated tandem solar cell. In Figure S21C, a direct comparison of tandem cell performance before HF cleaning and after the HF cleaning process is shown. Before the cleaning process, a degraded silver electrode with silver halide residues is attached directly on the metal chuck. After the cleaning process, additional silver metal paste is brushed on the cleaned region.

**Figure 3:**
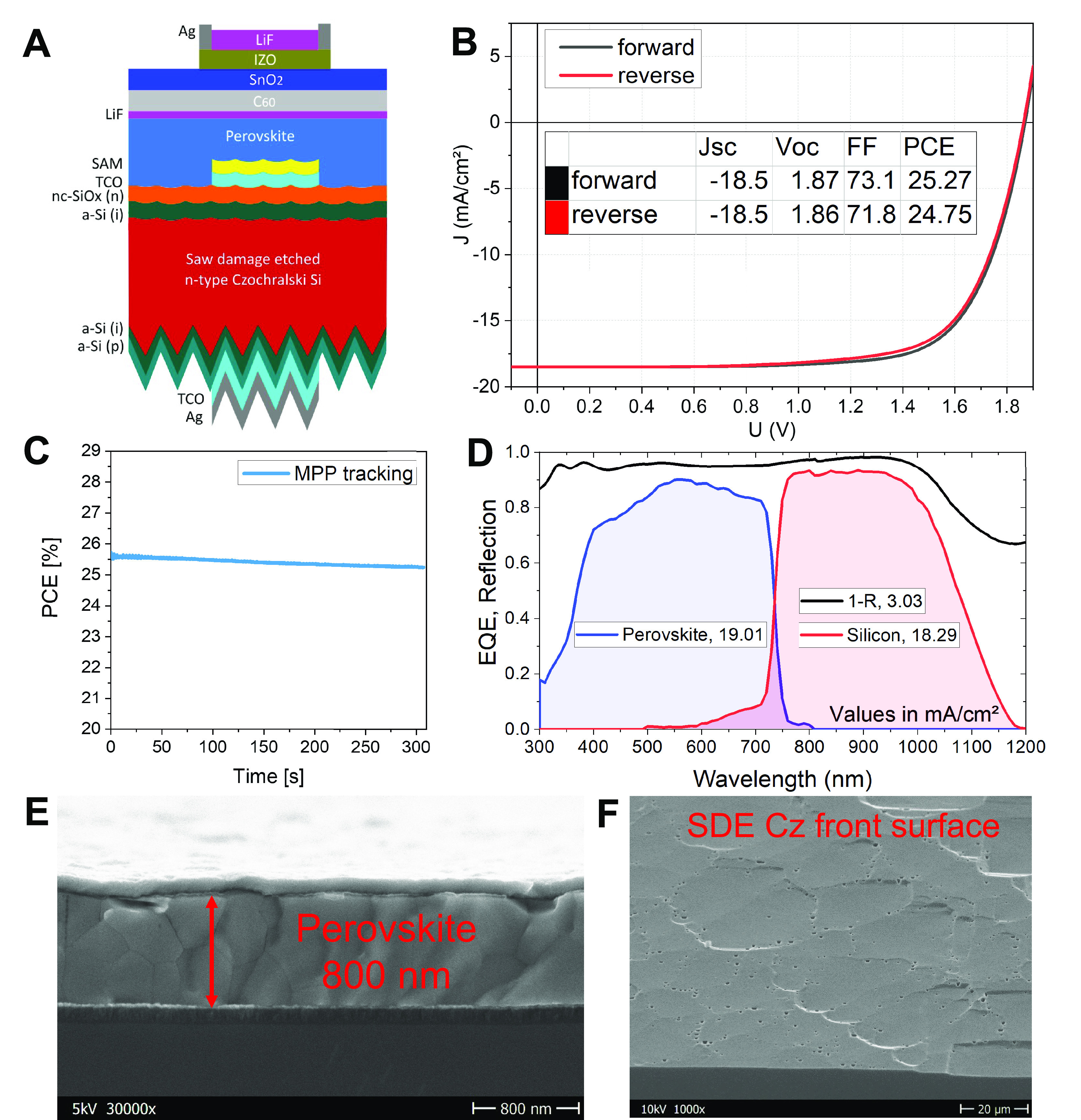
(A) Schematic of the two-terminal monolithic tandem layer stack developed in this work on a 120 μm saw damaged etched Czochralski silicon wafer with a rough front surface. The detailed layer stack is described in the Supporting Information. (B) *J*–*V* curves of the champion slot-die coated perovskite/silicon tandem solar cell. Red line: reverse scan (from *V*_oc_ to *J*_sc_); black line: forward scan. (C) MPP tracking over 300 s with stabilized PCE at 25.2%. (D) EQE and UV–vis (1-R, black line) measurements for the tandem solar cell. (E) Cross-sectional SEM images of a slot-die coated top cell. (F) 30° tilted SEM image of a saw damaged etched Czochralski silicon wafer front surface.

A schematic illustration of the slot-die coated tandem devices is shown in Figure 3A. The best tandem solar cell performance using the industrial, thin Cz wafer is shown in Figure 3B. This tandem solar cell reaches a PCE of 25.2% (*V*_oc_ = 1.87 V, *J*_sc_ = 18.5 mA/cm^2^, and FF = 73.1%) with an active area of 1 cm^2^ (see photographs of the active area layout in Figure S17). The corresponding stabilized PCE from the MPP tracks after 300 s is 25.2%, as shown in Figure 3C. To the best of our knowledge, the *V*_oc_ of 1.87 V is the highest yet reported value in a perovskite/Si tandem with a non-spin-coated perovskite absorber.

The tandem EQE spectra shown in Figure 3D validate our measured current density from the *J*–*V* curves (Figure 3B). In Figure 3D, the integration of the tandem EQE spectra gives a 19.01 mA/cm^2^ photocurrent for the perovskite absorber and a 18.29 mA/cm^2^ photocurrent for the silicon absorber. In order to have a clear comparison of the optical losses, in Figure S18, the spin coated 3cation on a thick FZ wafer is utilized as the reference EQE, which is deduced from ref (12) and compared in detail below. As seen in Figure 3F, the surface roughness of the SDE Cz bottom cell is rather large; however, the slot-die coating process has successfully covered the rough SDE surface when using an absorber thickness of over 800 nm (Figure 3E).

**Figure 4:**
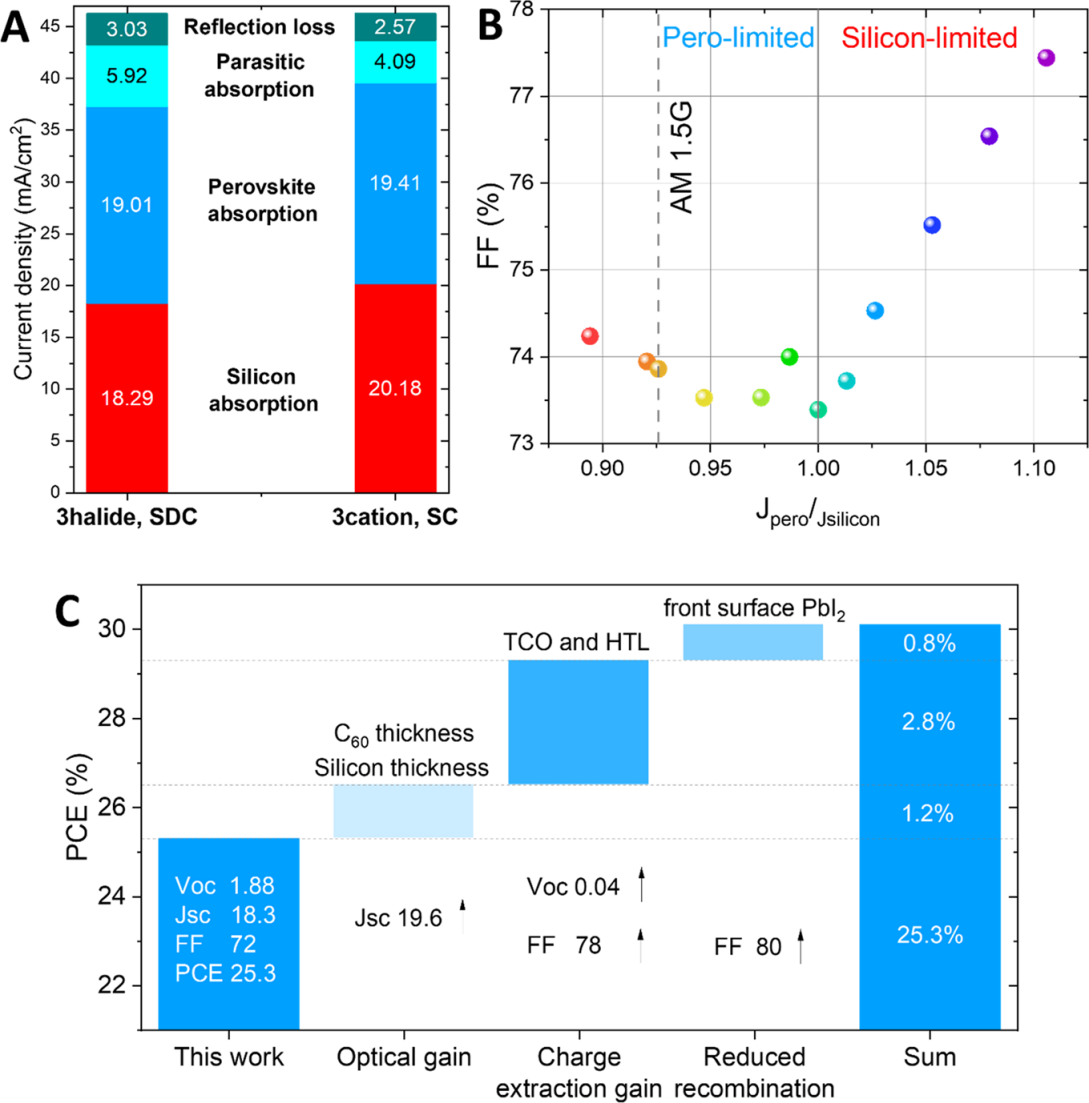
Efficiency loss analysis of slot-die coated perovskite silicon tandem solar cells. (A) Summary of current density losses in SDC perovskite/silicon tandem solar cell (this work) as extracted from Figure S18 in comparison to reported champion values using spin-coating and nonindustrial thick wafers.^12^ (B) Fill factor as a function of illumination with varied spectrum (red-rich to blue-rich) to induce current mismatch and thus determine the fill factor limitation of the bottom and top cells.^43^ (C) Loss analysis and roadmap toward 30%: a summary of optical and electrical losses for both the perovskite top and silicon bottom cell and the feasible solutions for further studies.

The best tandem solar cell developed in this work is still performing worse than the best reported tandem cells produced by spin-coating to fabricate the halide perovskites on thick floatzone (FZ) wafers. In order to shed light on the reasons for this difference, we perform a loss analysis here. In Figure S18, a direct comparison is made between the spin coated halide perovskites (so-called “3cation” with the following composition: Cs_0.05_(FA_0.77_MA_0.23_)_0.95_Pb(I_0.77_Br_0.23_)_3_) on FZ wafers with a thickness of 250 μm^12^ and the best slot-die coated 3halide perovskites on 120 μm thin Cz silicon bottom cells from this work. There are three main distinct differences: First, with 23 nm C_60_ for the SDC tandem instead of 18 nm C_60_ for SC, parasitic absorption between 300 and 600 nm is higher, as discussed previously.^17^ This C_60_ thickness used here is needed for enhancing solar cell stability and to make sure to cover this nonflat perovskite surface more conformally. Second, the thicker SDC halide perovskites film has a higher photocurrent and, with that, less transmission to the silicon bottom cell. As seen in the wavelength range from 600 to 730 nm, the halide perovskites gained 0.28 mA/cm^2^ photocurrent as compared to the spin coated film due to the 800 nm slot-die coated halide perovskites layer compared with the 600 nm spin coated layer thickness. Third, the insufficient absorption in the NIR wavelength (900–1200 nm) regime is caused by the thin silicon wafer.^44^ In addition, the detailed comparison for the two investigated layer stacks in Figure 4A and Figure S18 shows that parasitic absorption, being the difference between (1-reflection) and the sum of the perovskite and silicon EQE, is higher for the SDC cell, which could originate from higher absorption within the contact layers (the thicker C_60_ thickness with higher absorption). In the NIR range, the difference originates likely from the transparent conductive oxide (TCO) layers or the silicon bottom cell. Overall, by increasing the silicon wafer thickness of the bottom cell to above 200 μm or applying suitable front surface texture for light trapping and reduced parasitic absorption, we estimate a current matching value at around 19.6 mA/cm^2^, which would further enhance the PCE of the slot die coated tandem solar cell by 1.2% absolute (see Figure 4B).

In addition to the optical losses, we have also analyzed the FF limitation by measuring the cell performance under different spectral illumination conditions while keeping the same overall intensity as shown in Figure 4B. The measurement method has been discussed previously.^43^ Although the characterized sample shows lower perovskite photocurrent due to thin perovskite thickness, the mismatch trends still explain the tandem FF limitation. The generated current ratio has been utilized to saturate either the perovskite top cell or silicon bottom cell so that the specific subcell can no longer be FF limiting under specific illumination conditions. By increasing the infrared illumination for the silicon subcell, the perovskite-limited region reflects the perovskite dominated FF. On the contrary, by increasing the blue illumination for the perovskite subcell, the perovskite-limited region reflects the silicon dominated FF. With the asymmetrical FF shape of controlled current mismatch, it can be concluded that the FF limitation of the top cell still needs to be considered as an overall tandem FF limitation. Thus, the tandem FF of the best slot-die coated perovskite silicon tandem solar cell presented here is still limited by the perovskite top cell. As we have described here in the last section, the low charge carrier lifetime and PbI_2_ accumulated between the perovskite/C_60_ interface are likely the root issues to be investigated in a further study in more detail to improve the top cell fill factor further.

With the full tandem stack, we perform intensity-dependent *J*–*V* (suns-*V*_oc_) measurements,^39^ which are summarized in Figure S22. A pseudo fill factor of 80% is measured which forecasts the potential tandem performance. In comparison with the recently published 84% pFF of perovskite/silicon tandems constructed by selective electroluminescence (EL) measurements,^3^ the 4% pFF difference can be explained by limitations of the intrinsic perovskite bulk material which needs to be further improved with the targets of long lifetime and potential shunts reduction. In comparison between the suns-*V*_oc_ and *J*–*V* measurement in Figure 3B with 8% FF difference (averaged between the forward and reverse scans) and by the slopes of the *V*_oc_ intersect point, significant serial resistance and transport losses are still present in slot-die coated perovskite/silicon tandem solar cells presented here.

Higher fill factors could potentially be achieved when replacing the SAM molecule 2PACz with Me-4PACz,^12^ which has been known to improve charge extraction. The successful deployment of Me-4PACz for SDC 3halide could give significant PCE improvement by higher FF. Assuming the same improvements in FF here for slot die coated tandems and being able to transfer the single-junction *V*_oc_ into the tandem solar cells, a gain of 2.8% absolute efficiency is feasible.

Finally, the perovskites and electron transport layer (ETL) interface with accumulated PbI_2_ induce strong recombination loss and likely high resistance which should be investigated and be avoided in future research by proper interlayer or passivation strategies. Getting rid of this layer can further improve the FF to 80%, which has been proven for the spin coated 3halide single-junction (Figure 4C). Assuming a final tandem solar cell *V*_oc_ of 1.92 V, potentially 30.1% PCE could be generated by optimizing the slot-die coated top cell with our current knowledge. Further research and development, especially regarding the perovskites/C_60_ interface loss, will further improve the realistic efficiency potential and thus close the gap between slot die coated and spin coated perovskite/silicon tandem solar cells in the future.

We report on an efficient, wide bandgap triple-halide perovskite, with the composition of (Cs_0.22_FA_0.78_)Pb(I_0.85_Br_0.15_)_3_ + 5 mol % MAPbCl_3_, specifically for slot-die coating by developing a suitable solvent system. We demonstrate that, with these halide perovskites, our fabrication route enables a bandgap of 1.68 eV, which is suitable for tandem solar cells and without the phase segregation that is typically observed for high Br loadings. The slot-die coated wet perovskite films were dried using a stream of nitrogen (N_2_) from an “N_2_-knife” with high reproducibility and avoiding the need to use antisolvents. We find parameters allowing crystallization of the perovskite film into large grains reducing charge collection losses and, thus, enabling higher current density for the tandem solar cell. By annealing at 150 °C, an optimized trade-off between crystallization and the detrimental formation of PbI_2_ aggregates on the film’s top surface is found. Thus, we improve the cell stability and performance of halide perovskites single-junction cells toward a stabilized power output of up to 19.4%, and a two-terminal monolithic tandem solar cell with a PCE of 25.2% on a 1 cm^2^ active area is demonstrated with fully scalable processes. This tandem efficiency is reached for wafers with a thickness of around 120 μm, not yet enabling the full photocurrent potential in the NIR wavelength regime compared to thicker FZ wafers (>250 μm) that are typically used in the literature. Finally, we show a detailed comparison between spin coated and slot-die coated perovskite films. For the solar cells, we present the loss mechanisms as well as guidelines for further improving the printed films. With that, we highlight the high potential for slot-die coating as a fabrication route for scalable and industrially relevant perovskite/silicon tandem solar cells.

## Supporting Information Available

Experimental procedures, material and device fabrication, device characterization, PL spectra, XRD and GIXRD data, EDX of surface and cross section, intensity-dependent PL measurements, trPL measurements, SEM, PCEs for aged devices, TLM measurements, rear side protection, and intensity-dependent pseudo *J*–*V* measurement (PDF)

## Notes

**Data and Materials Availability.** All data are available in the main text or the Supporting Information.

## References

[ref1] Shockley W.; Queisser H. J. Detailed balance limit of efficiency of p–n junction solar cells. J. Appl. Phys. 1961, 32, 510–519. 10.1063/1.1736034.

[ref2] Richter A.; Hermle M.; Glunz S. W. Reassessment of the limiting efficiency for crystalline silicon solar cells. IEEE J. Photovoltaics 2013, 3, 1184–1191. 10.1109/JPHOTOV.2013.2270351.

[ref3] Tockhorn P., Berlin H., Sutter J., Berlin H., Berlin H.; Lang F. Nano-optical designs enhance monolithic perovskite/silicon tandem solar cells toward 29.8% efficiency. Nature portfolio 2022, 10.21203/rs.3.rs-1439562/v1, under review. PMC9646483 36280763

[ref4] Min H.; Lee D. Y.; Kim J.; Kim G.; Lee K. S.; Kim J.; Paik M. J.; Kim Y. K.; Kim K. S.; Kim M. G.; et al. Perovskite solar cells with atomically coherent interlayers on SnO2 electrodes. Nature 2021, 598, 444–450. 10.1038/s41586-021-03964-8. 34671136

[ref5] Gu E.; Tang X.; Langner S.; Duchstein P.; Zhao Y.; Levchuk I.; Kalancha V.; Stubhan T.; Hauch J.; Egelhaaf H. J.; et al. Robot-Based High-Throughput Screening of Antisolvents for Lead Halide Perovskites. Joule 2020, 4, 1806–1822. 10.1016/j.joule.2020.06.013.

[ref6] Remeika M.; Qi Y. Scalable solution coating of the absorber for perovskite solar cells. J. Energy Chem. 2018, 27, 1101–1110. 10.1016/j.jechem.2017.10.005.

[ref7] Kohlstädt M.; Yakoob M. A.; Würfel U. A Matter of Drying: Blade-Coating of Lead Acetate Sourced Planar Inverted Perovskite Solar Cells on Active Areas >1 cm^2^. Phys. Status Solidi Appl. Mater. Sci. 2018, 215, 1800419 10.1002/pssa.201800419.

[ref8] Chen B.; Yu Z. J.; Manzoor S.; Wang S.; Weigand W.; Yu Z.; Yang G.; Ni Z.; Dai X.; Holman Z. C.; et al. Blade coated Perovskites on Textured Silicon for 26%-Efficient Monolithic Perovskite/Silicon Tandem Solar Cells. Joule 2020, 4, 850–864. 10.1016/j.joule.2020.01.008.

[ref9] Subbiah A. S.; Isikgor F. H.; Howells C. T.; De Bastiani M.; Liu J.; Aydin E.; Furlan F.; Allen T. G.; Xu F.; Zhumagali S.; et al. High-performance perovskite single-junction and textured perovskite/silicon tandem solar cells via slot-die-coating. ACS Energy Lett. 2020, 5, 3034–3040. 10.1021/acsenergylett.0c01297.

[ref10] Dou B.; Whitaker J. B.; Bruening K.; Moore D. T.; Wheeler L. M.; Ryter J.; Breslin N. J.; Berry J. J.; Garner S. M.; Barnes F. S.; et al. Roll-to-Roll Printing of Perovskite Solar Cells. ACS Energy Lett. 2018, 3, 2558–2565. 10.1021/acsenergylett.8b01556.

[ref11] Hwang K.; Jung Y. S.; Heo Y. J.; Scholes F. H.; Watkins S. E.; Subbiah J.; Jones D. J.; Kim D. Y.; Vak D. Toward large scale roll-to-roll production of fully printed perovskite solar cells. Adv. Mater. 2015, 27, 1241–1247. 10.1002/adma.201404598. 25581092

[ref12] Al-Ashouri A.; Köhnen E.; Li B.; Magomedov A.; Hempel H.; Caprioglio P.; Márquez J. A.; Morales Vilches A. B.; Kasparavicius E.; Smith J. A.; et al. Monolithic perovskite/silicon tandem solar cell with > 29% efficiency by enhanced hole extraction. Science (80- 2020, 370, 1300–1309. 10.1126/science.abd4016. 33303611

[ref13] Hou Y.; Aydin E.; De Bastiani M.; Xiao C.; Isikgor F. H.; Xue D. J.; Chen B.; Chen H.; Bahrami B.; Chowdhury A. H.; et al. Efficient tandem solar cells with solution-processed perovskite on textured crystalline silicon. Science (80-.) 2020, 367, 1135–1140. 10.1126/science.aaz3691. 32139544

[ref14] Bush K. A.; Palmstrom A. F.; Yu Z. J.; Boccard M.; Cheacharoen R.; Mailoa J. P.; McMeekin D. P.; Hoye R. L. Z.; Bailie C. D.; Leijtens T.; et al. 23.6%-Efficient Monolithic Perovskite/Silicon Tandem Solar Cells With Improved Stability. Nat. Energy 2017, 2, 17009 10.1038/nenergy.2017.9.

[ref15] Bush K. A.; Manzoor S.; Frohna K.; Yu Z. J.; Raiford J. A.; Palmstrom A. F.; Wang H. P.; Prasanna R.; Bent S. F.; Holman Z. C.; et al. Minimizing Current and Voltage Losses to Reach 25% Efficient Monolithic Two-Terminal Perovskite-Silicon Tandem Solar Cells. ACS Energy Lett. 2018, 3, 2173–2180. 10.1021/acsenergylett.8b01201.

[ref16] Schwenzer J. A.; Hellmann T.; Nejand B. A.; Hu H.; Abzieher T.; Schackmar F.; Hossain I. M.; Fassl P.; Mayer T.; Jaegermann W.; et al. Thermal Stability and Cation Composition of Hybrid Organic-Inorganic Perovskites. ACS Appl. Mater. Interfaces 2021, 13, 15292–15304. 10.1021/acsami.1c01547. 33764733

[ref17] Xu J.; Boyd C. C.; Yu Z. J.; Palmstrom A. F.; Witter D. J.; Larson B. W.; France R. M.; Werner J.; Harvey S. P.; Wolf E. J.; et al. Triple-halide wide-band gap perovskites with suppressed phase segregation for efficient tandems. Science (80-.) 2020, 367, 1097–1104. 10.1126/science.aaz5074. 32139537

[ref18] Jošt M.; Köhnen E.; Morales-Vilches A. B.; Lipovšek B.; Jäger K.; Macco B.; Al-Ashouri A.; Krč J.; Korte L.; Rech B.; et al. Textured interfaces in monolithic perovskite/silicon tandem solar cells: Advanced light management for improved efficiency and energy yield. Energy Environ. Sci. 2018, 11, 3511–3523. 10.1039/C8EE02469C.

[ref19] Slotcavage D. J.; Karunadasa H. I.; McGehee M. D. Light-Induced Phase Segregation in Halide-Perovskite Absorbers. ACS Energy Lett. 2016, 1, 1199–1205. 10.1021/acsenergylett.6b00495.

[ref20] Petrov A. A.; Ordinartsev A. A.; Fateev S. A.; Goodilin E. A.; Tarasov A. B. Solubility of hybrid halide perovskites in DMF and DMSO. Molecules 2021, 26, 7541 10.3390/molecules26247541. 34946624 PMC8706401

[ref21] Li J.; Dagar J.; Shargaieva O.; Többens D.; Munir R.; Unger E. 20.8% slot-die coated MAPbI3 perovskite solar cells by optimal DMSO-content and age of 2-ME based precursor inks. Adv. Energy Mater. 2021, 11, 2003460 10.1002/aenm.202003460.

[ref22] Hendriks K. H.; Van Franeker J. J.; Bruijnaers B. J.; Anta J. A.; Wienk M. M.; Janssen R. A. J. 2-Methoxyethanol as a new solvent for processing methylammonium lead halide perovskite solar cells. J. Mater. Chem. A 2017, 5, 2346–2354. 10.1039/C6TA09125C.

[ref23] Kim M.; Kim G. H.; Lee T. K.; Choi I. W.; Choi H. W.; Jo Y.; Yoon Y. J.; Kim J. W.; Lee J.; Huh D.; et al. Methylammonium Chloride Induces Intermediate Phase Stabilization for Efficient Perovskite Solar Cells. Joule 2019, 3, 2179–2192. 10.1016/j.joule.2019.06.014.

[ref24] Al-Ashouri A.; Magomedov A.; Roß M.; Jošt M.; Talaikis M.; Chistiakova G.; Bertram T.; Márquez J. A.; Köhnen E.; Kasparavičius E.; et al. Conformal monolayer contacts with lossless interfaces for perovskite single junction and monolithic tandem solar cells. Energy Environ. Sci. 2019, 12, 3356–3369. 10.1039/C9EE02268F.

[ref25] Ava T. T.; Al Mamun A.; Marsillac S.; Namkoong G. A review: Thermal stability of methylammonium lead halide based perovskite solar cells. Appl. Sci. 2019, 9, 188 10.3390/app9010188.

[ref26] Lee J. W.; Dai Z.; Lee C.; Lee H. M.; Han T. H.; De Marco N.; Lin O.; Choi C. S.; Dunn B.; Koh J.; et al. Tuning Molecular Interactions for Highly Reproducible and Efficient Formamidinium Perovskite Solar Cells via Adduct Approach. J. Am. Chem. Soc. 2018, 140, 6317–6324. 10.1021/jacs.8b01037. 29723475

[ref27] Yi C.; Luo J.; Meloni S.; Boziki A.; Ashari-Astani N.; Grätzel C.; Zakeeruddin S. M.; Röthlisberger U.; Grätzel M. Entropic stabilization of mixed A-cation ABX3 metal halide perovskites for high performance perovskite solar cells. Energy Environ. Sci. 2016, 9, 656–662. 10.1039/C5EE03255E.

[ref28] Park B.-w.; Seok S. I. Intrinsic Instability of Inorganic–Organic Hybrid Halide Perovskite Materials. Adv. Mater. 2019, 31, 1805337 10.1002/adma.201805337. 30773706

[ref29] Kim M.; Lee T. K.; Choi I. W.; Choi H. W.; Jo Y.; Lee J.; Kim G.-H.; Kwak S. K.; Kim D. S. Effects of cation size and concentration of cationic chlorides on the properties of formamidinium lead iodide based perovskite solar cells. Sustain. Energy Fuels 2020, 4, 3753 10.1039/D0SE00382D.

[ref30] Jeon N. J.; Noh J. H.; Yang W. S.; Kim Y. C.; Ryu S.; Seo J.; Seok S. Il Compositional engineering of perovskite materials for high-performance solar cells. Nature 2015, 517, 476–480. 10.1038/nature14133. 25561177

[ref31] Rabbani A.; Ayatollahi S. Comparing three image processing algorithms to estimate the grain-size distribution of porous rocks from binary 2D images and sensitivity analysis of the grain overlapping degree. Spec. Top. Rev. Porous Media 2015, 6, 71–89. 10.1615/SpecialTopicsRevPorousMedia.v6.i1.60.

[ref32] Shargaieva O.; Lang F.; Rappich J.; Dittrich T.; Klaus M.; Meixner M.; Genzel C.; Nickel N. H. Influence of the Grain Size on the Properties of CH3NH3PbI3 Thin Films. ACS Appl. Mater. Interfaces 2017, 9, 38428–38435. 10.1021/acsami.7b10056. 29039197

[ref33] Rehermann C.; Merdasa A.; Suchan K.; Schröder V.; Mathies F.; Unger E. L. Origin of Ionic Inhomogeneity in MAPb(IxBr1-x)3Perovskite Thin Films Revealed by In-Situ Spectroscopy during Spin Coating and Annealing. ACS Appl. Mater. Interfaces 2020, 12, 30343–30352. 10.1021/acsami.0c05894. 32510922

[ref34] Krückemeier L.; Rau U.; Stolterfoht M.; Kirchartz T. How to Report Record Open-Circuit Voltages in Lead-Halide Perovskite Solar Cells. Adv. Energy Mater. 2020, 10, 1902573 10.1002/aenm.201902573.

[ref35] Stolterfoht M.; Wolff C. M.; Márquez J. A.; Zhang S.; Hages C. J.; Rothhardt D.; Albrecht S.; Burn P. L.; Meredith P.; Unold T.; et al. Visualization and suppression of interfacial recombination for high-efficiency large-area pin perovskite solar cells. Nat. Energy 2018, 3, 847–854. 10.1038/s41560-018-0219-8.

[ref36] Staub F.; Hempel H.; Hebig J. C.; Mock J.; Paetzold U. W.; Rau U.; Unold T.; Kirchartz T. Beyond Bulk Lifetimes: Insights into Lead Halide Perovskite Films from Time-Resolved Photoluminescence. Phys. Rev. Appl. 2016, 6, 044017 10.1103/PhysRevApplied.6.044017.

[ref37] Caprioglio P.; Caicedo-Dávila S.; Yang T. C. J.; Wolff C. M.; Peña-Camargo F.; Fiala P.; Rech B.; Ballif C.; Abou-Ras D.; Stolterfoht M.; et al. Nano-emitting Heterostructures Violate Optical Reciprocity and Enable Efficient Photoluminescence in Halide-Segregated Methylammonium-Free Wide Bandgap Perovskites. ACS Energy Lett. 2021, 6, 419–428. 10.1021/acsenergylett.0c02270.

[ref38] Herterich J.; Unmüssig M.; Loukeris G.; Kohlstädt M.; Würfel U. Ion Movement Explains Huge VOC Increase despite Almost Unchanged Internal Quasi-Fermi-Level Splitting in Planar Perovskite Solar Cells. Energy Technol. 2021, 9, 2001104 10.1002/ente.202001104.

[ref39] Stolterfoht M.; Grischek M.; Caprioglio P.; Wolff C. M.; Gutierrez-Partida E.; Peña-Camargo F.; Rothhardt D.; Zhang S.; Raoufi M.; Wolansky J.; et al. How To Quantify the Efficiency Potential of Neat Perovskite Films: Perovskite Semiconductors with an Implied Efficiency Exceeding 28. Adv. Mater. 2020, 32, 2000080 10.1002/adma.202000080. 32163652

[ref40] Caprioglio P.; Wolff C. M.; Sandberg O. J.; Armin A.; Rech B.; Albrecht S.; Neher D.; Stolterfoht M. On the Origin of the Ideality Factor in Perovskite Solar Cells. Adv. Energy Mater. 2020, 10, 2000502 10.1002/aenm.202000502.

[ref41] Lang F.; Kohnen E.; Warby J.; Xu K.; Grischek M.; Wagner P.; Neher D.; Korte L.; Albrecht S.; Stolterfoht M. Revealing Fundamental Efficiency Limits of Monolithic Perovskite/Silicon Tandem Photovoltaics through Subcell Characterization. ACS Energy Lett. 2021, 6, 3982–3991. 10.1021/acsenergylett.1c01783.

[ref42] Svanström S.; Jacobsson T. J.; Boschloo G.; Johansson E. M. J.; Rensmo H.; Cappel U. B. Degradation Mechanism of Silver Metal Deposited on Lead Halide Perovskites. ACS Appl. Mater. Interfaces 2020, 12, 7212–7221. 10.1021/acsami.9b20315. 31958007

[ref43] Köhnen E.; Jošt M.; Morales-Vilches A. B.; Tockhorn P.; Al-Ashouri A.; Macco B.; Kegelmann L.; Korte L.; Rech B.; Schlatmann R.; et al. Highly efficient monolithic perovskite silicon tandem solar cells: Analyzing the influence of current mismatch on device performance. Sustain. Energy Fuels 2019, 3, 1995–2005. 10.1039/C9SE00120D.

[ref44] Köhnen E.; Wagner P.; Lang F.; Cruz A.; Li B.; Roß M.; Jošt M.; Morales-Vilches A. B.; Topič M.; Stolterfoht M.; et al. 27.9% Efficient Monolithic Perovskite/Silicon Tandem Solar Cells on Industry Compatible Bottom Cells. Sol. RRL 2021, 5, 2100244 10.1002/solr.202100244.

