# Supplementary material for: Slot-Die Coated Triple-Halide Perovskites for Efficient and Scalable Perovskite/Silicon Tandem Solar Cells
Source: ACS Energy Lett. 2022 Sep 27;7(10):3600–11. doi: 10.1021/acsenergylett.2c01506 (PMC9578656; doi:10.1021/acsenergylett.2c01506)
Supplement: Supplementary file 1 — nz2c01506_si_001.pdf [file nz2c01506_si_001.pdf]

Supplementary Information for

Slot-die coated triple-halide perovskites for efficient and scalable perovskite/silicon tandem solar cells

Ke Xu, Amran Al-Ashouri, Zih-Wei Peng, Eike Köhnen, Hannes Hempel, Fatima Akhundova, Jose A. Marquez, Philipp Tockhorn, Oleksandra Shargaieva, Florian Ruske, Jiahuan Zhang, Janardan Dagar, Bernd Stannowski, Thomas Unold, Daniel Abou-Ras, Eva Unger[*]*, Lars Korte, Steve Albrecht*[*]

*Corresponding author Email: steve.albrecht@helmholtz-berlin.de, eva.unger@helmholtz-berlin.de

This PDF file includes:

Experimental Procedures

Device characterization

Supplementary Data

The investigation of the reproducibility issue of the slot-die coated perovskite silicon solar cell

Figure S1 to S22

Tables S1 to S2

References

Experimental Procedures

Materials

Name	Full Name/ extra description	Company
Anhydrous DMSO	dimethyl sulfoxide, 99.9%	Sigma Aldrich
Anhydrous DMF	Dimethylformamide, 99.8%	Sigma Aldrich
NMP	1-Methyl-2-pyrrolidinone, 99.5%	Sigma Aldrich
LiF	lithium fluoride (purity ⩾ 99.99%)	Sigma Aldrich
MACl	Methylamonium chloride, 99%	Greatcell Solar
C_{60} (sublimed)	sublimed	CreaPhys GmbH
FAI	formamidinium iodide, 99.9%	Dyenamo
CsI	Cesium Iodide, 99.999%	abcr GmbH
IZO	Indium Zinc Oxide	FHR Anlagenbau GmbH
PbI_2	Lead Iodide, 99.99%	TCI
$PbBr_2$	Lead Bromide, 99.99%	TCI
$PbCl_2$	Lead Chloride, 99%	TCI: #L0291
2PACz	[2-(9H-carbazol-9-yl)ethyl]phosphonic acid	TCI
Ethanol	Anhydrous absolute Ethanol	VWR

Perovskite ink formation

The triple-halide perovskite composition used in the present study was $Cs_{0.22}FA_{0.78}Pb(I_{0.85}Br_{0.15})_3$ (denoted as CS22Br15) + 5 mol% $MAPbCl_3$. For each mol Cs22Br15, 0.05 mol of $MAPbCl_3$ extra was added to form the total salt. Here we used a 1.4 mol/ml solution, which means 1 mol total salt is diluted in 50 µl NMP and 664 µl DMF stirring at room temperature (30°C) to fully solubilize.

Perovskite single-junction preparation

ITO-covered glass (Automated Research, 15 Ω/sq sheet resistance) was used for the deposition of our inverted (p-i-n) planar structure perovskite solar. During the cleaning process, Mucasol solution (2% in DI-Water), DI-water, acetone and isopropanol were used each for 15 min in a room temperature ultrasonic bath. Then the ITO substrates were activated for 15 min in an UV-O_3 cleaner (FHR UVOH 150 Lab) which was very vital for the coating of 2PACz layer ([2-(9H-carbazol-9-yl)ethyl]phosphonic acid). The single-junction cell configuration was ITO/HTL/Perovskite/LiF/ C_{60} /(BCP, SnO_2)/(Ag, Cu), where the HTL was 2PACz. The spin coated SAM layer and slot-die coated perovskite layer deposition steps were conducted in a nitrogen atmosphere without any exposure to air.

The hole transport material 2PACz (3 mmol, or ~1 mg/ml, in Ethanol absolute) was deposited using spin-coating (5 s acceleration to 3000 rpm, 3000 rpm for 25 s), followed by heating for 10 min at 100°C.

The perovskite solution was prepared as mentioned above. The slot-die coating machine (FOM Technologies, FOM ALPHASC) was equipped with slot-die head with a 2 cm shim width, which was 50 µm thick shim (FOM Technologies, Research Series). The air knife (Nex Flow, 10012XHA) is equipped with 0.5 mm nominal thickness shim. Coating details are listed below:

Coating process: the shim protruding the slot-die head was positioned 0.3-0.4 mm above the substrate to be coated, referred to as the coating *gap* between the slot-die head and substrate. After positioning the die head above the substrate, the precursor solution was pumped into the slot-die head to form a meniscus in between slot-die head and substrate. Then the coating process was started. The slot-die head was moved at speed of 5 mm/s with 300 µl/min pump rate while keeping the coating table and glovebox environment at 21°C. Otherwise, voids can be formed between perovskite and substrate interface leading to coating inhomogeneities illustrated in Figure S3.

Drying process and annealing process: after coating the wet film, the chuck was moved right beneath the air knife which was supplied with 22-24 psi of N_2, being located 1-1.5 cm above the substrate, perpendicular pointing towards the surface. The velocity of the substrate moving under the *air knife* was, as for the coating step, 5 mm/s. We moved the pre-dried film onto the hot plate for annealing under various annealing temperatures, for the duration of 20 min in a N_2-filled glovebox.

Afterwards, 23 nm of C_{60} was thermally evaporated at a rate of 0.15 Å s^{-1} onto the perovskite film. As indicated, a 1 nm-thick LiF interlayer was deposited between C_{60} and perovskite, evaporated at a rate of 0.05 Å s^{-1}, within the same vacuum run as the C_{60} layer. A thermal atomic layer deposition ALD Arradiance GEMStar reactor was used to deposit 20 nm SnO_2. The Sn Precursor was Tetrakis (dimethylamion)tin(IV) (TDMASn) which was held at 60°C in a stainless-steel container. We used water as oxidant, which was passed from a stainless-steel container without active heating. With the deposition temperature of 80°C. The continuous 140 cycles accumulated 20 nm tin oxide. As an alternative, 8 nm BCP, which could be evaporated at 0.15 Å s^{-1} onto the C_{60} film, had the same function as SnO_2. At the end, either 100 nm Silver (Ag) or 100 nm Copper (Cu) were also deposited with the rate of 1 Å s^{-1}.

Silicon solar cell preparation (Czochralski)

The fabrication details of our silicon heterojunction (SHJ) cells with rear side p/n junction can be found in [1]**.** Just briefly:

The n-type Czochralski (Cz) wafer, with <100> orientation and 140 µm thickness as well as 5 Ω·cm resistivity, was cleaned and chemically treated for saw damage etching removal and one-side texturing with random pyramids as described in [2]. In order to remove the surface residual oxide, the c-Si wafers were cleaned by RCA procedures and dipped in 1% hydrofluoric acid for minutes. For the 105 µm thick wafer which is used for the session of the slot-die coated perovskite/silicon tandem reproducibility investigation (see Figure S21A), it has an additional front side chemical polishing. Then the intrinsic and doped silicon films were deposited by plasma-enhanced chemical vapor deposition on both sides. An i/p stack (junction) and i/n stack (front surface field) were deposited on the rear and front side, respectively. To enhance light coupling into the bottom cell we used the same nanocrystalline silicon oxide n-layer stack as an optical interlayer [1]. Both front (20 nm) and the rear (150 nm) TCO layers were DC sputtered from a commercial TCO target (nSCOT) at room temperature. 400 nm Ag layer was applied as the rear metallization by sputtering for rear metallization. Both contact-layer stacks of the silicon were deposited using aligned shadow masks with an opening of 1.13×1.13 cm^2. After fabrication, the silicon bottom cell had been annealed at 210°C for 10 min before perovskite top cell deposition.

Preparation of perovskite/silicon for tandem solar cells

The silicon bottom cells were washed with ethanol by spin coating and cleaned for 15 min in an UV-O_3 cleaner (FHR UVOH 150 Lab). The same coating process, methods, and steps of HTL, perovskites, LiF, C_{60} and SnO_2 were deposited as described above on the silicon bottom cells. In the next step, 100 nm IZO was deposited by sputtering (Roth&Rau MicroSys 200 PVD, ceramic target purchased from FHR Anlagenbau GmbH). The 2-inch ceramic target consisted of 90%wt. In_2O_3 and 10%wt. ZnO. We applied 70W RF oscillation power to form a uniform deposition with 0.2%vol gas inlet O_2 in the chamber. The optimized layer had mobility, carrier density and resistivity of 43.5 $cm^2 V^{-1} s^{-1}$, 3.4·1020 cm^{-3} and $4.2 \cdot 10^{-4}$ Ω cm, respectively [3]. Subsequently, a 100 nm thick silver frame was thermally evaporated as bus bar on the boundary of the active area to collect the charge carriers. At last, 100 nm LiF was also thermally evaporated as an antireflective coating.

Device characterization

Time-dependent steady-state photoluminescence

The time-dependent steady-state absolute photoluminescence measurements were performed on a home-made setup including a 532 nm laser, and an integrating sphere. The samples were placed at the sphere edge. The PL signal was collected through an optical fiber towards a CCD-array spectrometer (Ocean Optics). The samples were excited by a continuous-wave laser at 532 nm. The photon flux was around 1.2×10^{16} photons/s with a spot size of 0.12 cm^2. This intensity was around 1 sun excitation flux which has been calibrated with a certified silicon reference cell. The spot size was measured by fitting a Gaussian curve to the beam profile extracted from the CCD imaging. Non-absorbed laser light and emitted photoluminescence fluxes were collected by the spectrometer. The PLQY could be calculated by the ratio of the laser absorption and emitted photoluminence. The PL signal was recorded with an integration time of 300 ms with a delay between measurements of one second, and the result was averaged over 10 measurements. Measurements were carried out in air ambient immediately after taking the samples out from the N_2-filled glovebox.

Absolute PL and intensity dependent QFLS measurements

520 nm CW laser (Insaneware) was utilized for the PL excitation light source through an optical fiber into an integrating sphere. The light source intensity has been calibrated into 1 sun condition by the active area of 1 cm^2 perovskite solar cell. The current density under short-circuit condition matched the Jsc under the sun simulator (1.375×10^{22} photons $m^{-2} s^{-1}$). An independent optical fiber was utilized as the output of the integrating sphere to a spectrometer (Andor SR393i-B) which was equipped with silicon CCD camera (DU420A-BR-DD, iDus). Halogen lamp with known spectral irradiance was utilized to calibrate the whole system. Between the spectral output of the detector and the calibrated spectral irradiance of the lamp, a spectral correction factor was utilized. The spectral photon density was calculated from the corrected detector signal by division of the photon energy. Thus, the photon excitation and emission numbers were received from numerical integration by Matlab. At last, three test samples with high known PLQY from Hamamatsu Photonics were utilized to accurately compare and reproduce within a small relative error of less than 5%.

During the measurement, the samples were placed under illumination into the integrating sphere using the same 520 nm CW laser which was described before. To control the intensity, a continuously variable neutral density filter wheel (ThorLabs) was utilized to attenuate the laser input to measure at different intensities. The intensity was also recorded by the additional silicon detectors. The illuminated samples experienced for a variable illumination time using an electrical shutter for a specific intensity. After 1 second illumination time, the PL spectra were received by averaging 30 spectra taken using a 30 µs exposure time by a detector. The electrical shutter was closed, and the filter wheel rotated to a new position. The measurements were repeated and recorded in this way. A home-made LabView code was written to automate the measurement and automate the data evaluation.

Pseudo J-Vs

In previous publication [4], the pseudo J-V has been introduced from intensity-dependent QFLS or V_{oc} measurements. At a specific light intensity which can also be converted into equivalent intensities as compared to ''suns'', this method calculated the dark-current density from the generated current density. For example, for the wide bandgap 1.68 eV perovskite, 1 sun corresponds to 20.5 mA/cm². 1% of 1 sun corresponds to 0.205 mA/cm². In specific illumination intensity, the measured QFLS or V_{oc} were measured to create a transport/series resistance free dark J-V curve. Then, the curve ws shifted to the corresponding J_{sc} under certain sun ratios. This method allows to derive pseudo-FF of the partial cell stack, such as bare perovskite film or whole solar cells. Under such investigation, the implied FF is impacted purely by the non-radiative recombination processes without charge transport or resistive losses by the bulk material or the transport layers. Steady-state intensity dependent V_{oc} measurement was measured with a Wavelabs Sinus-70 LED class AAA sun simulator. By tuning the illumination intensity proportionally, the corresponding J-V scans were recorded. The construction method is similar to the explanation before.

Transient photoluminescence

The transient photoluminescence measurements were performed in confocal setup using a pulsed laser at 125 kHz repetition rate and center wavelength of 655 nm. The photon flux per excitation pulse was adjusted to approximately $3x10^{11}$ cm^{-2}, which should induce a carrier concentration that is comparable to steady-state conditions under solar illumination. For detection a single photon counting system with a Geiger-mode avalanche photodiode was used. The laser was filtered out by a 700 nm long pass filter.

Single junction solar cell characterization (current-voltage curves, EQE)

The J-V measurements of single-junction solar cells were recorded in the nitrogen atmosphere with the solar simulator (Oriel LCS-100) and Keithley 2400 source-measured unit. The measurement system was controlled by a LabView program. The sun simulator was calibrated to AM1.5G as 1 sun equivalent with a filtered KG3 Silicon reference solar cell which has been calibrated by Fraunhofer ISE. The spectral mismatch was around 0.997 within the measurement error. J-V scans were performed in a 2-point-probe configuration with a step size of 20 mV, an integration time of 20 ms and a settling time of 20 ms (250 mV/s). There is no pretreatment of the perovskite solar cells before the measurement. All the J-V

curve measurements were taken several times for better statistics. Shunted devices, showing significantly lower V_{oc} values were not considered in the statistical analysis.

EQE spectra were measured with an Oriel Instruments QEPVSI-b system with a Newport 300W Xenon arc lamp, utilizing the TracQ-Basic software. A calibrated silicon reference cell was applied before every measurement to calibrate the light intensity. The electrical response of the device under illumination was recorded with a Stanford Research SR830 Lock-In simplifier with the time constant of 0.3 s and evaluated in TracQ. We generally find good agreement between the integrated external quantum efficiency times AM1.5G irradiance and the J_{sc} from J-V scan values.

Tandem solar cell characterization (current-voltage curves, EQE)

A Wavelabs Sinus-70 LED class AAA sun simulator was used for the measurement of the tandem solar cells in air condition under AM1.5G (1sun) equivalent illumination. No preconditioning of the tandem solar cells was applied before measurement. With the current density obtained by EQE, the limited junction can be determined. The intensity was changed slightly by sample to sun simulator distance so that the Jsc of the limited sub cell can be reached.

The backside of the solar cell was contacted onto a metal vacuum chuck at 25°C. On the front side, two Au probes contacted the front silver electrode. A metal laser-cut aperture mask covered the substrate with the exposure area as an active area. The J-V measurement and MPP tracking were recorded using a home-build LabView software. The EQE spectra were recorded with a homemade setup by using 78 Hz chopped monochromatic light from a Xe and He light source respectively. For the measurement of perovskite top junction EQE, the silicon bottom cell was saturated by using an 850 nm peak emission LED. In order to maintain the short circuit conditions, a 0.6V bias voltage was applied. For the silicon junction EQE, the silicon bottom cell EQE was measured by saturating the perovskite top cell by 455 nm blue LED and an additional 1 V bias voltage.

XRD

X-ray diffraction patterns (XRD) were measured using a Bruker D8 diffractometer in Bragg-Brentano geometry with a custom-made inert sample holder at room temperature. For all measurements, the Cu K-alpha radiation emitted from an X-ray tube operated at 40 mA and 40 kV acceleration voltage was used.

GIXRD

Grazing incidence X-ray diffraction (GIXRD) measurements were carried out using a Bruker D8 diffractometer. The height of the films was calibrated before each measurement. Diffraction patterns were collected with a step size of 0.02 degrees, for 5 seconds at each step, with the measurement conducted in air.

UV-VIS

For transmission and reflection measurements a PerkinElmer Lambda – 1050 UV/VIS/NIR spectrophotometer with an integrating sphere was used.

Grain size estimation

The SEM images have been processed by MATLAB script which has been modified for our images' adaption [5].

Supplementary Data

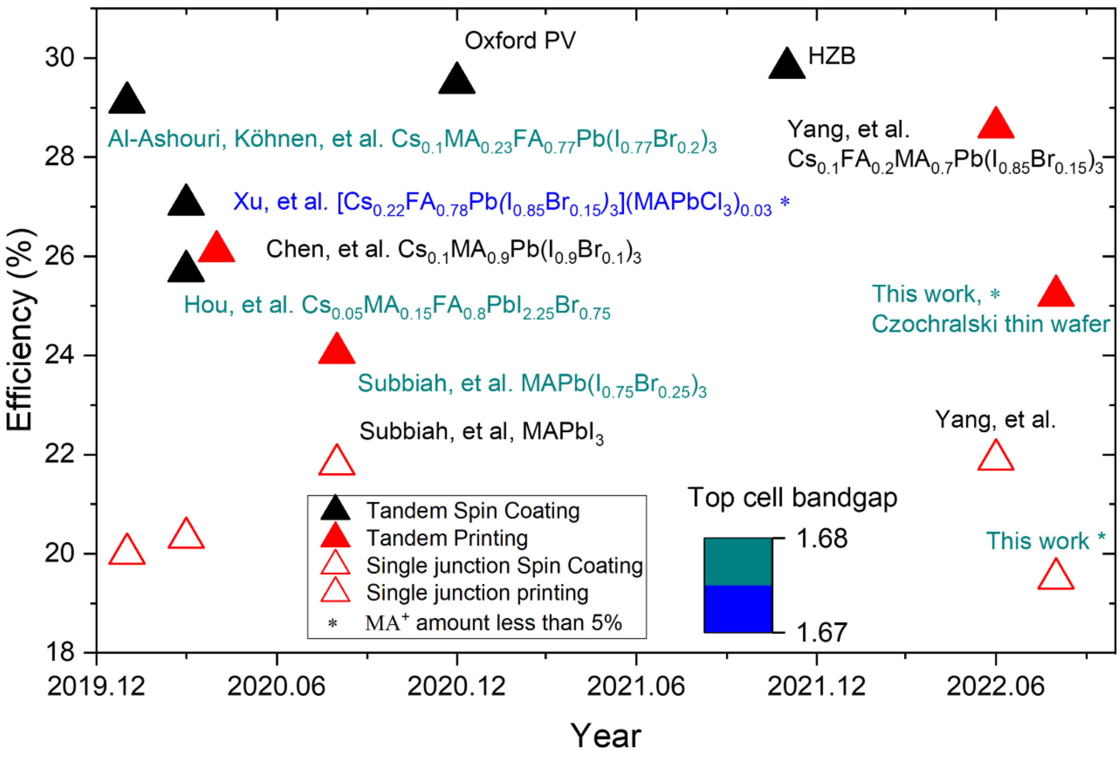

Figure S1. Efficiency vs. time for state-of-the-art perovskite single junction and perovskite/silicon tandem cells with various coating techniques and perovskite precursors. The coating techniques included spin coating, inkjet printing, blade coating and slot-die coating. There was no inkjet printed perovskite for tandem solar cells. For publications 5 and 6, the corresponding single-junction was shown for comparison without extra label and information. All data is collected below in the Table S1. Cells with low methylammonium iodide (MAI) concentration have been highlighted with *.

Table S1. Literature overview of state of the art single and tandem solar cells using various coating techniques and perovskite precursors, as shown in Figure S1.

Time	Tandem (PCE)	Single cell (top cell) PCE	Bandgap (eV)	MA^+ (%)	Coating methods	Publication
2020.01	29.1	20.8	1.68	23	Spin coating	Monolithic perovskite/silicon tandem solar cell with > 29% efficiency by enhanced hole extraction [6]
2020.03	25.7		1.68	15	Spin coating	Efficient tandem solar cells with solution-processed perovskite on textured crystalline silicon [7]
2020.03	27.04	20.32	1.67	3	Spin coating	Triple-halide wide–band gap perovskites with suppressed phase segregation for efficient tandems [8]
2020.04	26.1		Unknown	90	Blade coating	Blade-Coated Perovskites on Textured Silicon for 26%-Efficient Monolithic Perovskite/Silicon Tandem Solar Cells [9]
2020.08	24.05	21.8	1.68	100	Slot-die coating	High-Performance Perovskite Single-Junction and Textured Perovskite/Silicon Tandem Solar Cells via Slot-Die Coating [10]
2020.12	29.5		Unknown		Unknown	Oxford PV
2021.11	29.8		1.66	21	Spin coating	HZB
2022.04		19.5	1.68	5	Slot-die coating	This work
2022.08	25.2		1.68	5	Slot-die coating	This work
2022.06	28.6	21.9	1.65	70	Blade-coating	Defect engineering in wide-bandgap perovskites for efficient perovskite–silicon tandem solar cells

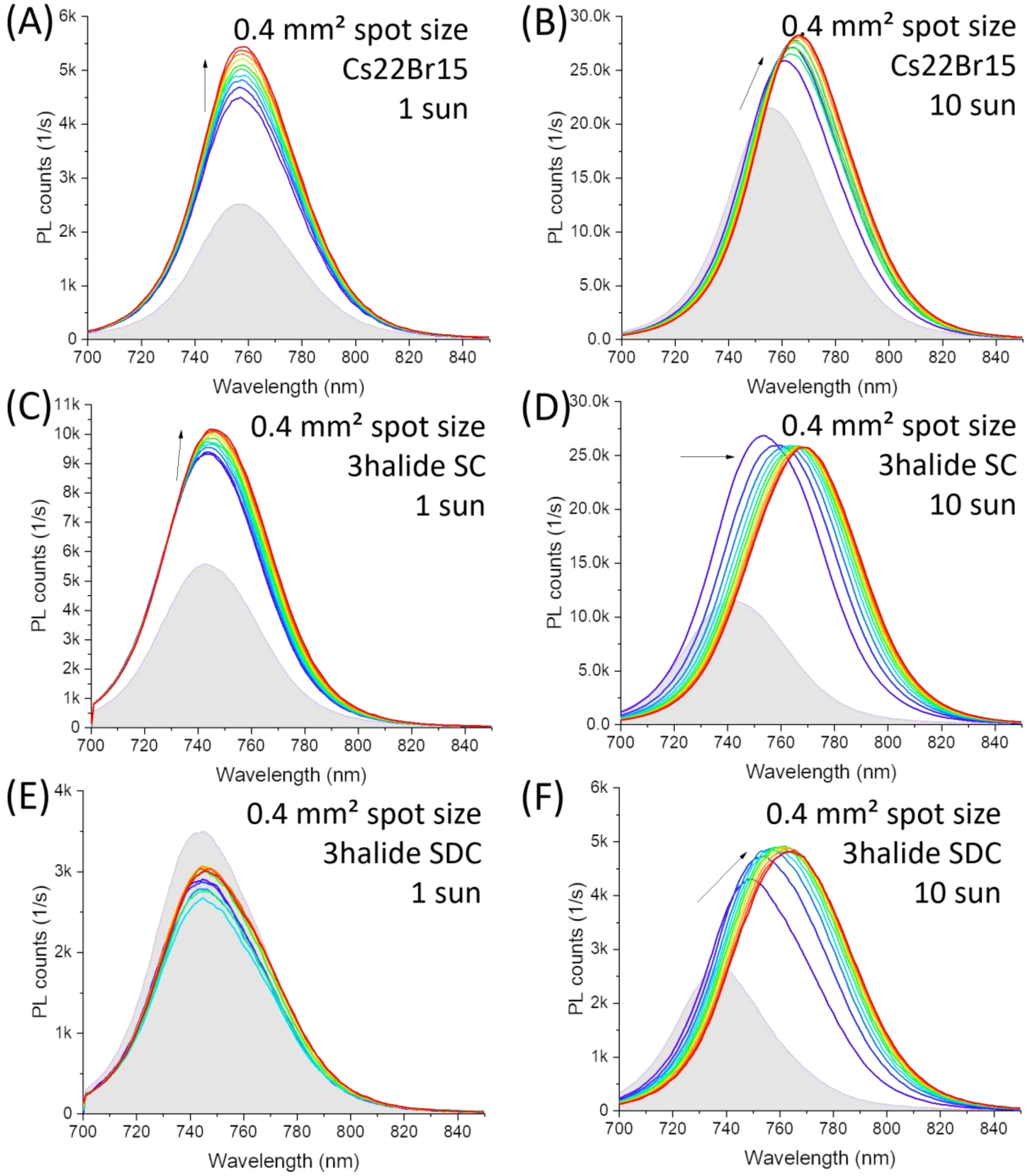

Figure S2. Photoluminescence of perovskite on ITO/2PACz substrates as a function of time for various excitation intensities over 150 seconds. The initial PL intensity is described as the gray area. The color shifts from blue to red with 0 second to 150 seconds. The spot size is 0.4 mm² at 1 sun or 10 sun equivalent excitation fluence to examine the photoinduced phase segregation: (A) and (B), spin coated Cs22Br15 double cation perovskite under 1 sun and 10 sun illumination. (C) and (D), spin coated 3halide under 1 sun and 10 sun illumination. (E) and (F), slot-die coated 3halide under 1 sun and 10 sun illumination.

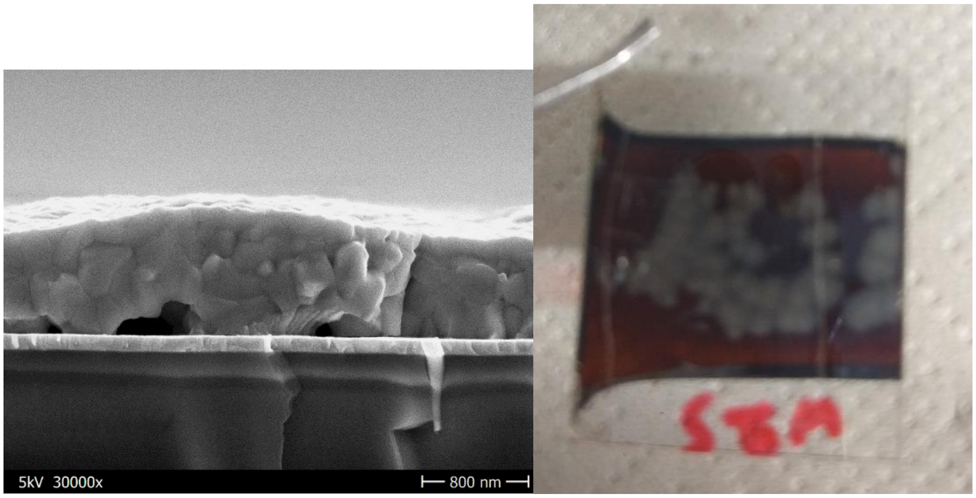

Figure S3. Left: SEM image showing that perovskite layer detaches from the substrate at 30°C coating table temperature. Right: Photograph of the slot-die coated ITO glass. The image was recorded from the glass side. The white region corresponds to the detached region in the left SEM image, at the interface of perovskite and ITO.

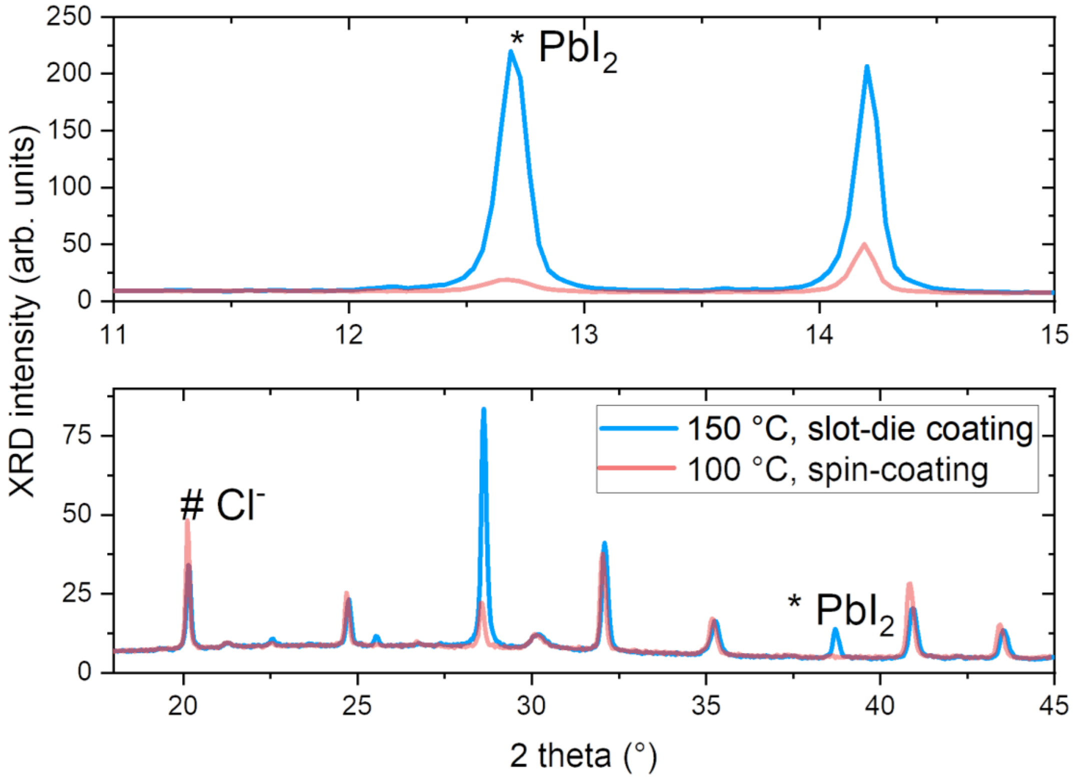

Figure S4: Angular X-ray diffraction (XRD) patterns for spin-coated 3halide perovskite composition annealed at 100°C for 20 mins. The PbI_2-to-perovskite peak ratio is significantly lower than for the slot-die coated samples annealed at 150°C (or higher temperatures, not shown here).

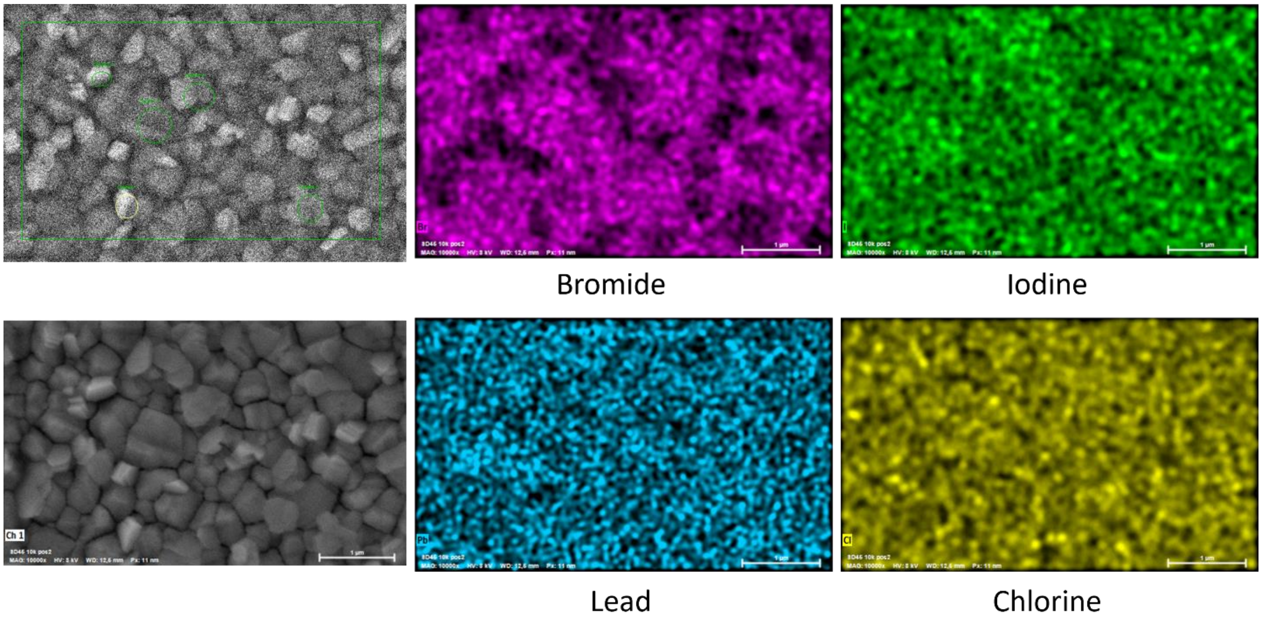

Figure S5. The EDX top view image with the annealing condition of 150°C for slot-die coating. Bromide, which is the only element, shows distribution inhomogeneity.

Table S2. The compositional EDX statistic of the top surface is listed in the table with 7 elements for 3 annealing conditions. The decrement of the Chloride trend follows the same bandgap increment. The lead concentration trend can also be observed. In the low energy band 0.5 keV, the iodine shows higher signal while the 3.9 keV shows no difference. The iodine statistic increment is contributed from the surface. It draws our attention towards the surface compositional investigation. The data was collected by the scanning area of 20 μm^2 in total.

Spectrum	C (%)	N (%)	Cl (%)	Br (%)	I (%)	Cs (%)	Pb (%)
170°C	19	15	1	7	41	0	18
150°C	19	16	1	8	39	0	16
100°C	19	18	2	7	37	1	16

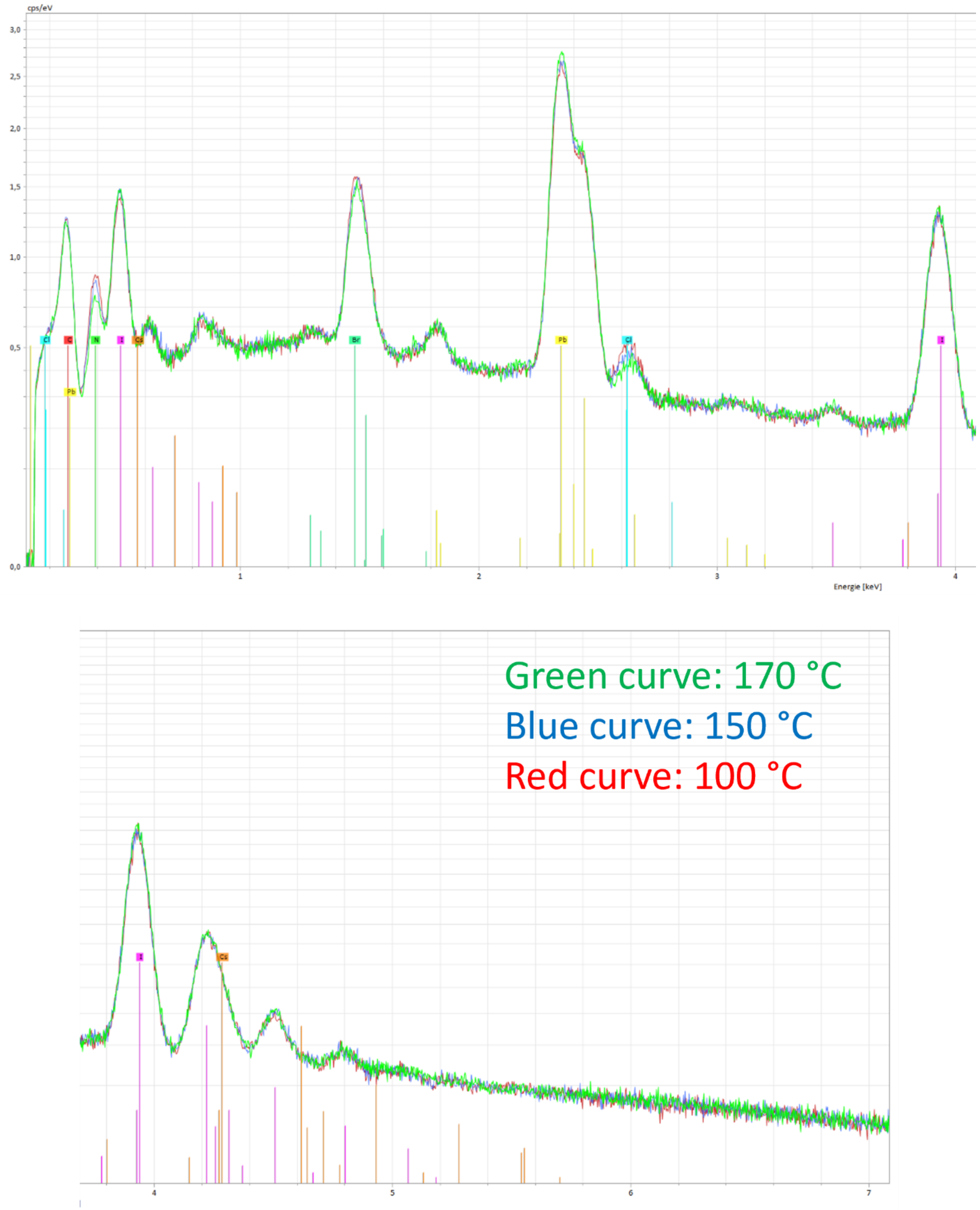

Figure S6. The original surficial EDX measurement data for 3 annealing conditions. The scanning range is from 0 to 7 keV.

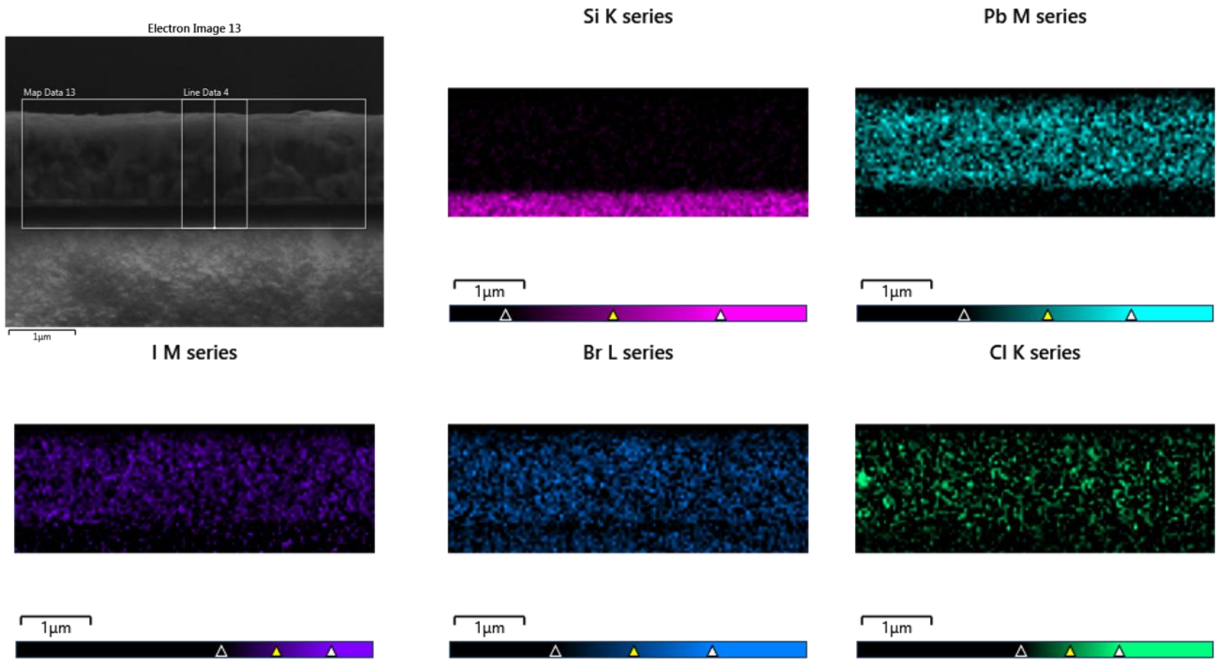

Figure S7. The compositional cross-view EDX measurement displaying with 5 different elements (silicon, lead, iodide, bromide and chloride) for perovskite films with 100°C annealing condition. The silicon graph is shown to highlight the boundary between the ITO and perovskite interface.

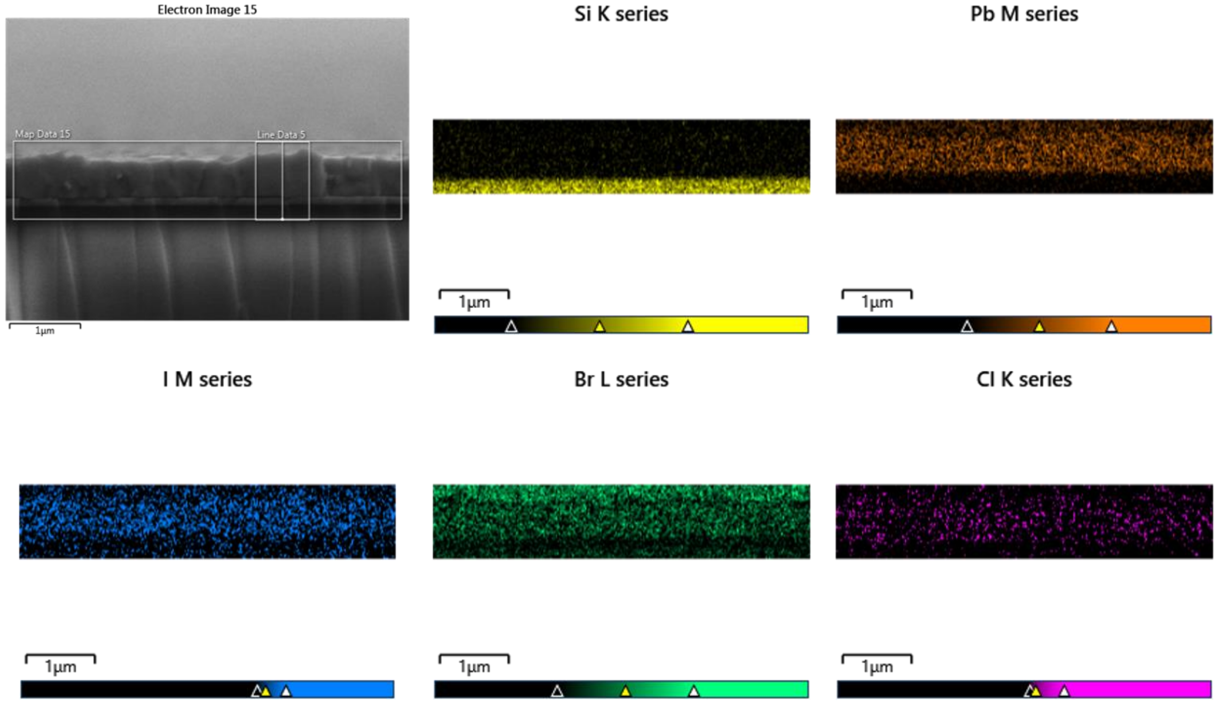

Figure S8. The compositional cross-view EDX measurement with five elements (silicon, lead, iodide, bromide and chloride) with 150°C annealing condition. The silicon graph is shown to highlight the

boundary between the ITO and perovskite interface. A clear Bromide gradient could be observed towards the surface with higher concentration.

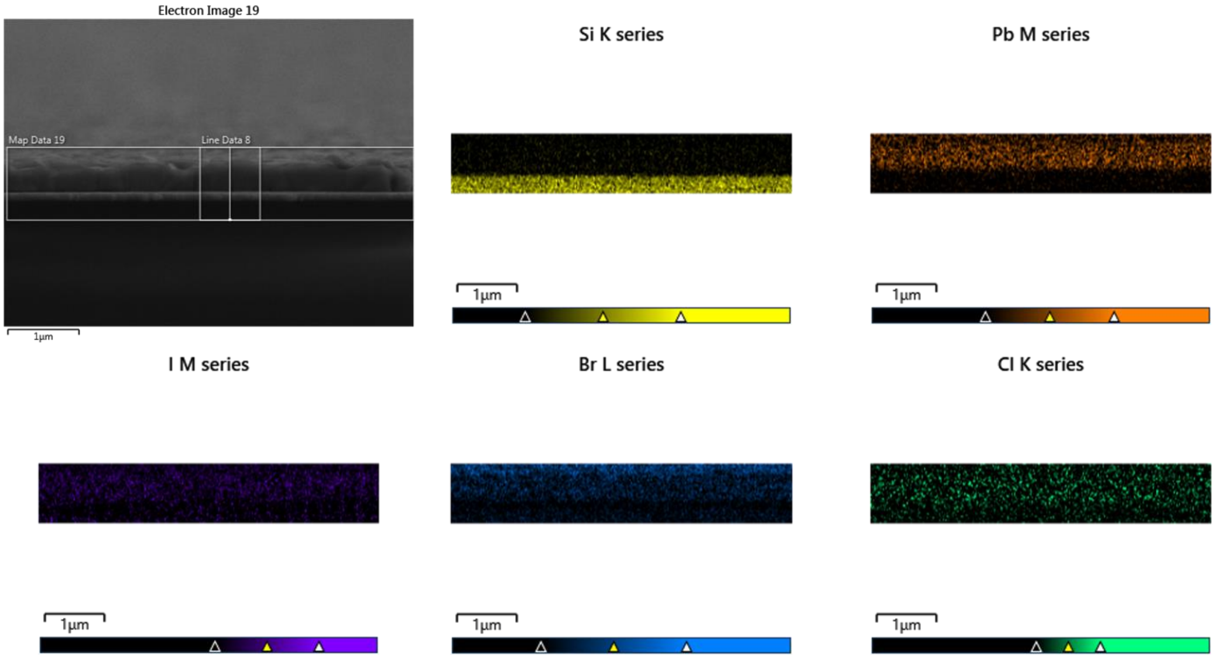

Figure S9. The compositional cross-view EDX measurement with five elements (silicon, lead, iodide, bromide and chloride) with 170°C annealing condition. The silicon graph is shown to highlight the boundary between the ITO and perovskite interface.

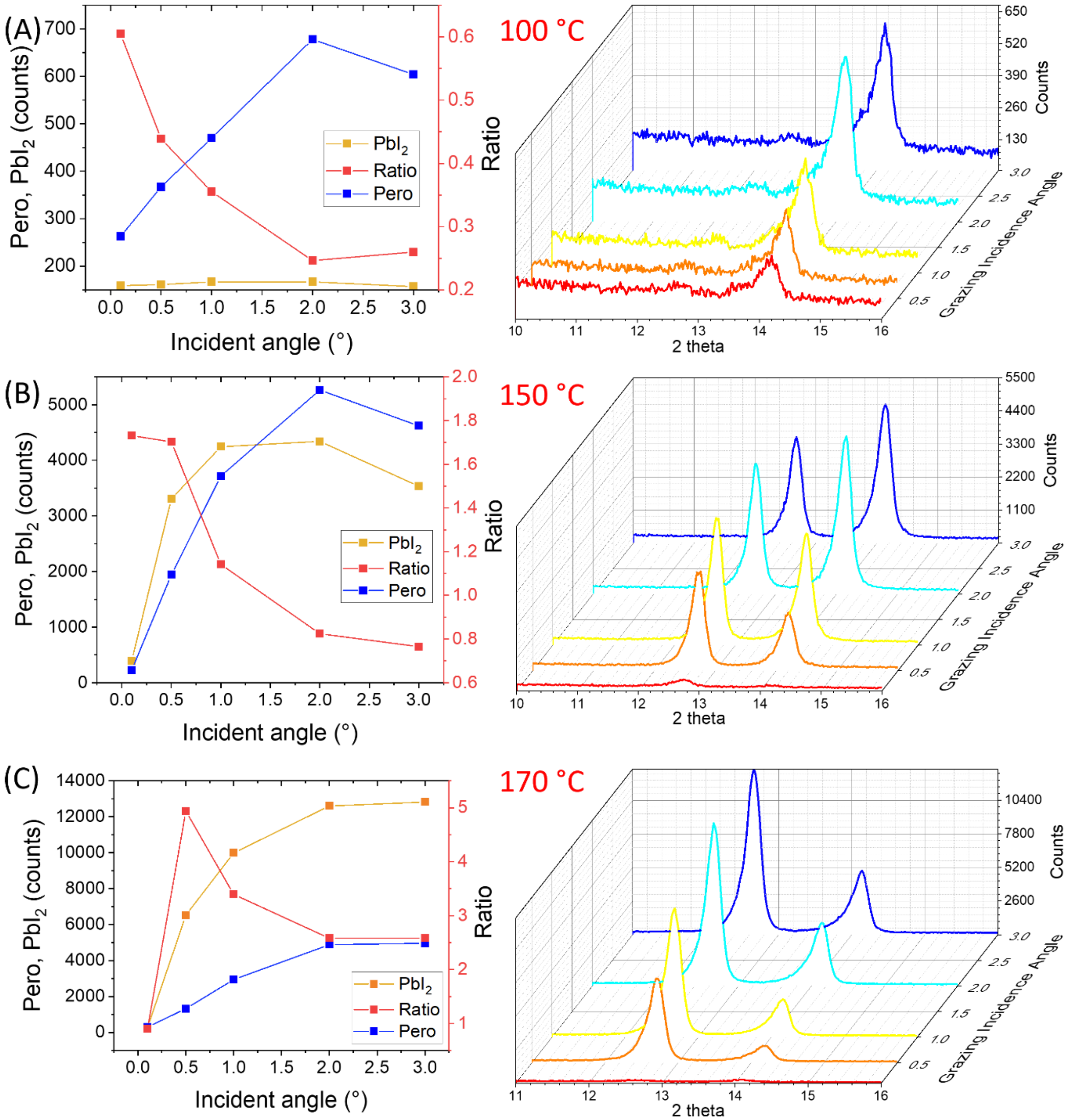

Figure S10. GIXRD, surface composition detection under three annealing conditions, (A) 100°C, (B) 150°C, (C) 170°C. The ratio between PbI_2 peak and perovskite peak explains that the PbI_2 is accumulated mainly on the surface when the annealing temperature raised to 170°C and it turns out to form another additional layer to influence the charge transportation.

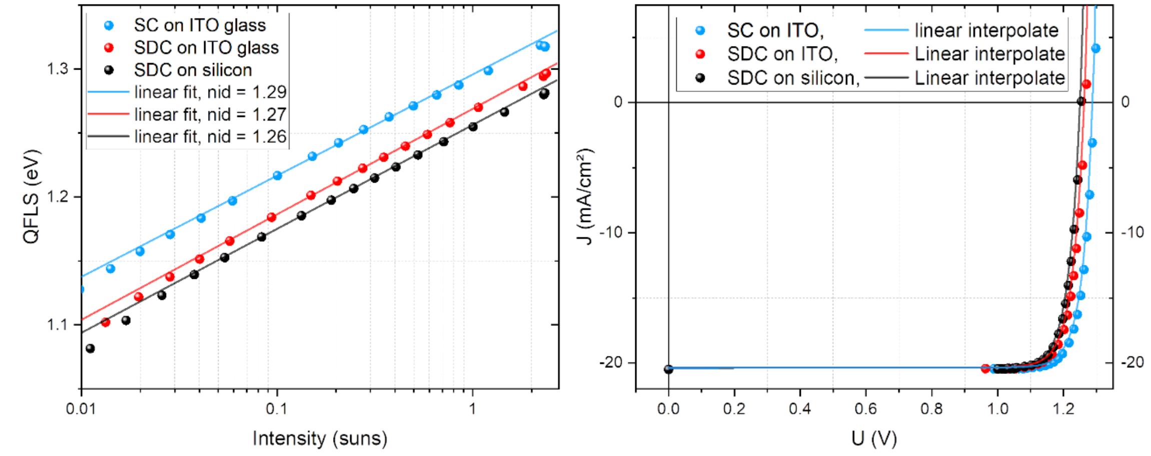

Figure S11. (A) QFLS as calculated from illumination-dependent PL [4] on a log-lin scale. The ideality factor measurement for various coating conditions is calculated from the fitted line slopes. SC denotes spin coating, SDC denotes slot-die coating. Because of the improved passivation between the substrate and perovskite interface, the PLQY shifts to higher values on quartz glass, as compared to ITO/glass. (B) The regressed pseudo J-V curves with the data points calculated corresponding current density. In order to calculate the pV_{oc}, the J_o values have been calculated by the method which has been discussed in previous work [4]. The spin coated perovskite film gives $J_o = 1.1 \times 10^{-21}$ A/m² and the slot-die coated perovskite film gives 1.8×10^{-21} A/m². From this curve, it is possible to derive the pVoc, pFF, pPCE which have been summarized in Table 1.

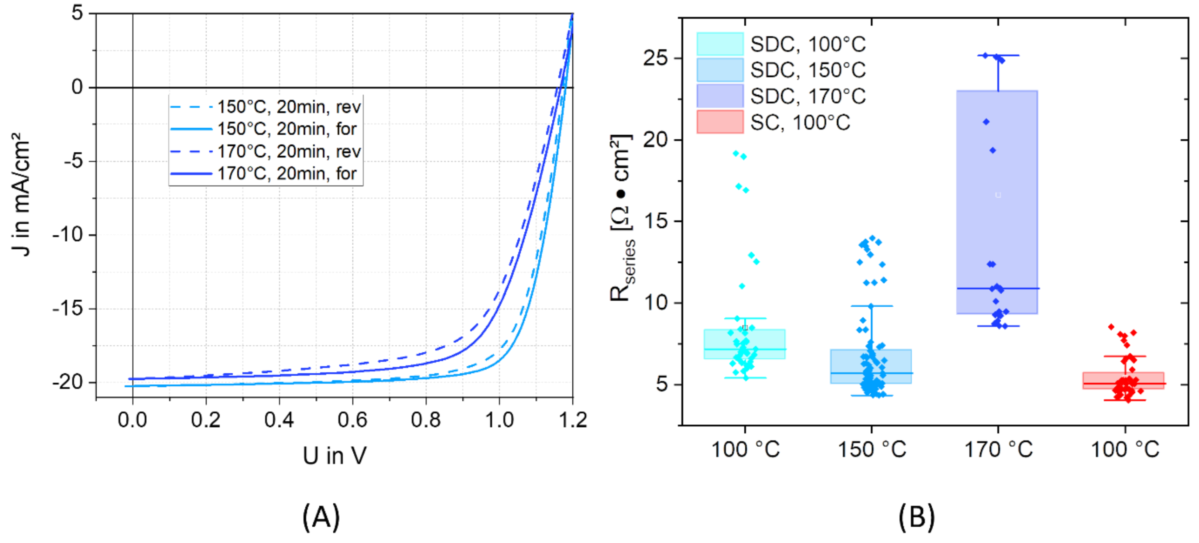

Figure S12. (A) J-V characterization of two annealing conditions. The series resistance increases significantly for the 170°C annealed solar cells. (B) extracted series resistance from Figure 2E at the slope

of the V_{oc} intersect points. The additional spin coated 3halide single junction series resistance has been added for comparison as red dataset.

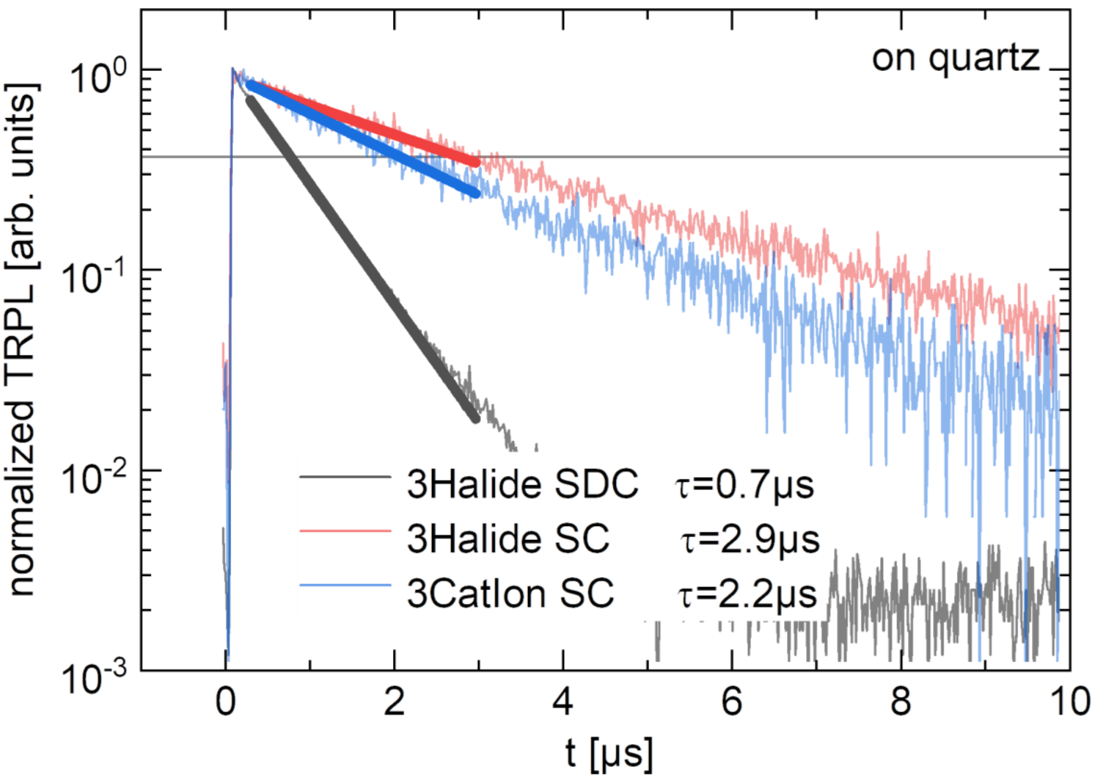

Figure S13. trPL measurement on quartz glass, with the same measurement condition. The slot-die coated 3halide perovskites gave 0.7 µs carrier lifetime on quartz glass while the spin coated 3halide perovskites give 2.9 µs. The difference comes mainly from the nonradiative recombination. The 3cation recipe is $(CsI)_{0.05}[FA_{0.77}MA_{0.23}Pb(I_{0.77}Br_{0.23})_3]_{0.95}$ [6].

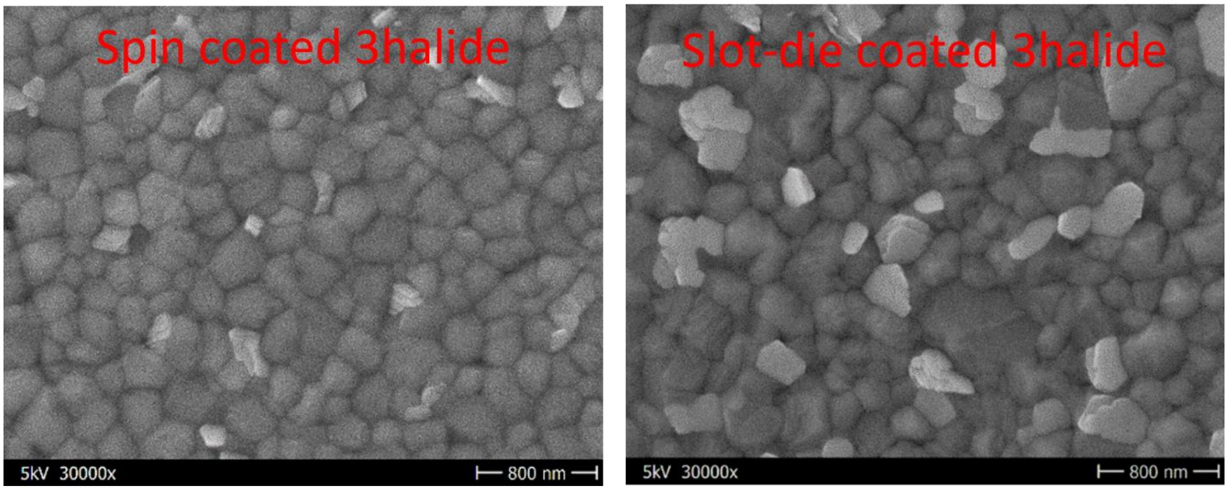

Figure S14. SEM top-view images for 3halide perovskites films using both 1.4M solution: left: spin coated 3halide film on ITO glass, right: slot-die coated 3halide film on ITO coated Cz substrates.

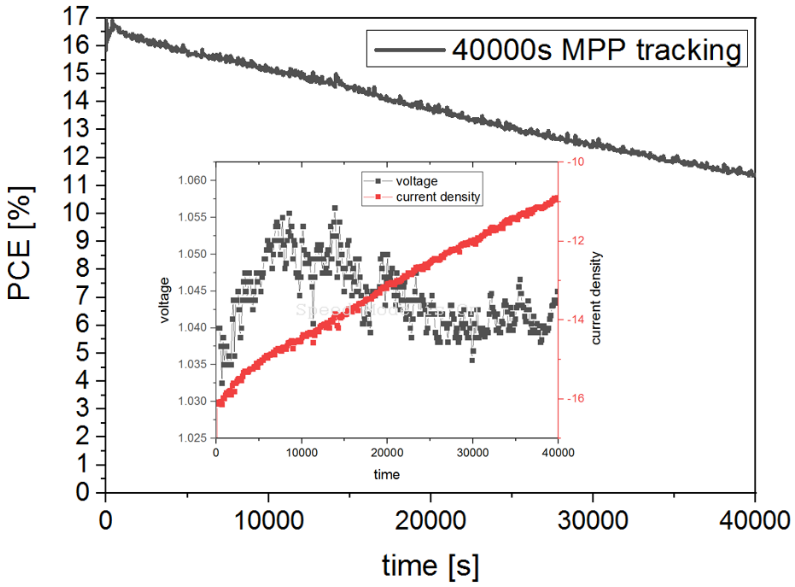

Figure S15. 40000 seconds MPP tracking for single junction solar cell with 100°C annealing condition by slot-die coating. The inset graph is the detailed Voc and Jsc tracking overtime.

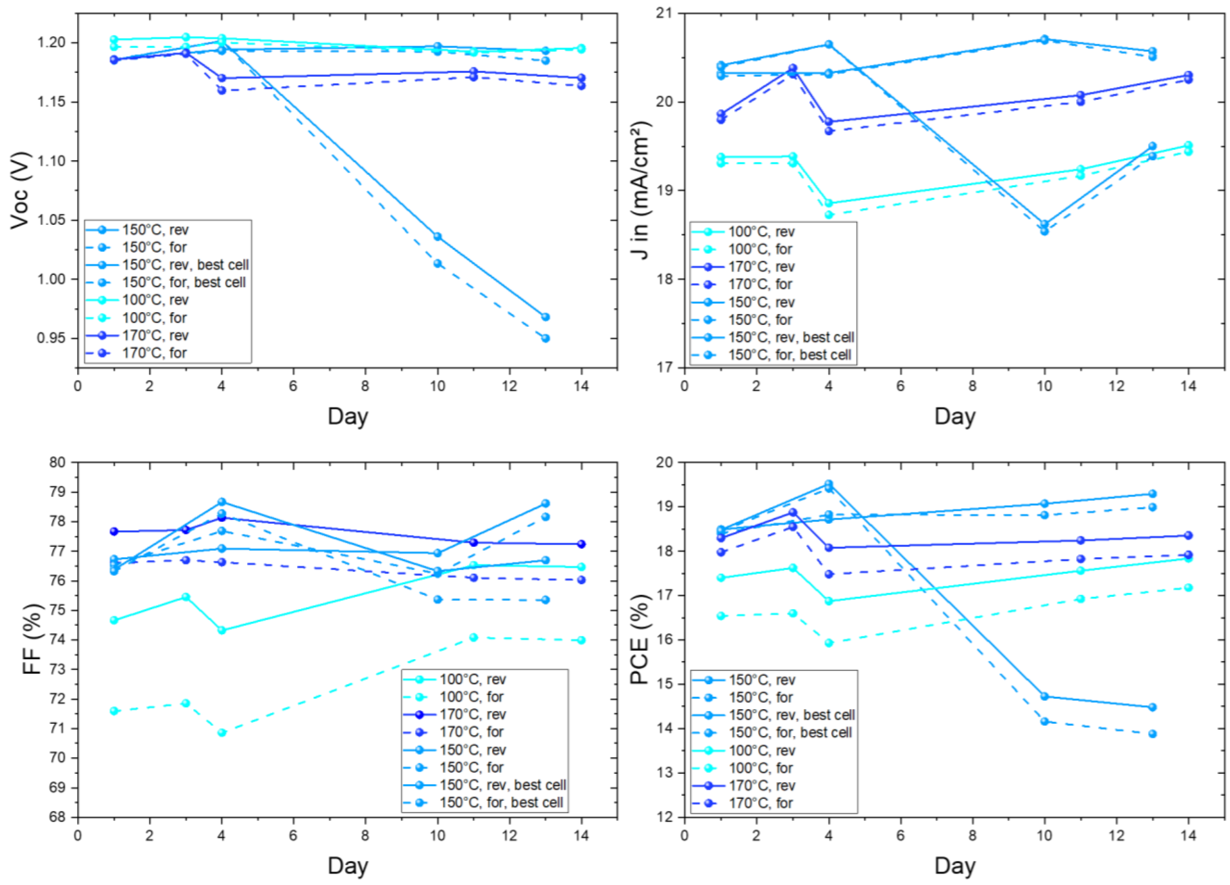

Figure S16. stability measurement over 14 days for the comparison of 3 annealing conditions. The best cell has been used for MPP tracking under the light illumination for 13 hours. The continuous illumination has destroyed the 150°C annealed best cell after day 4.

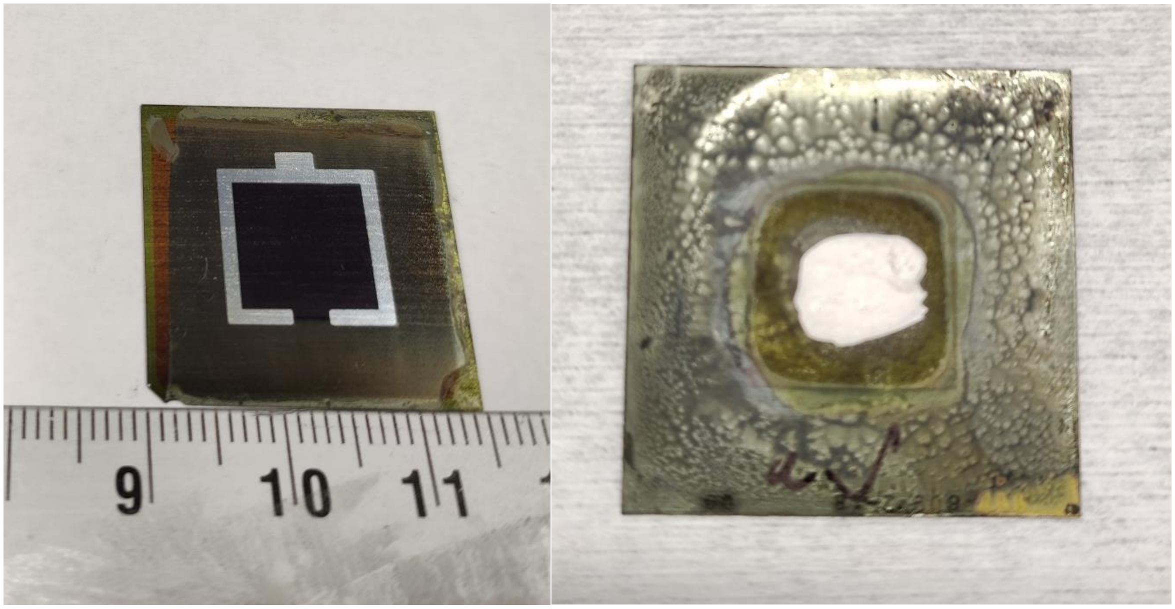

Figure S17. Left: Perovskite silicon tandem cell within the silver ring region. The measurement with a mask of 1.008 cm². Right: rear electrode brushed with silver paste.

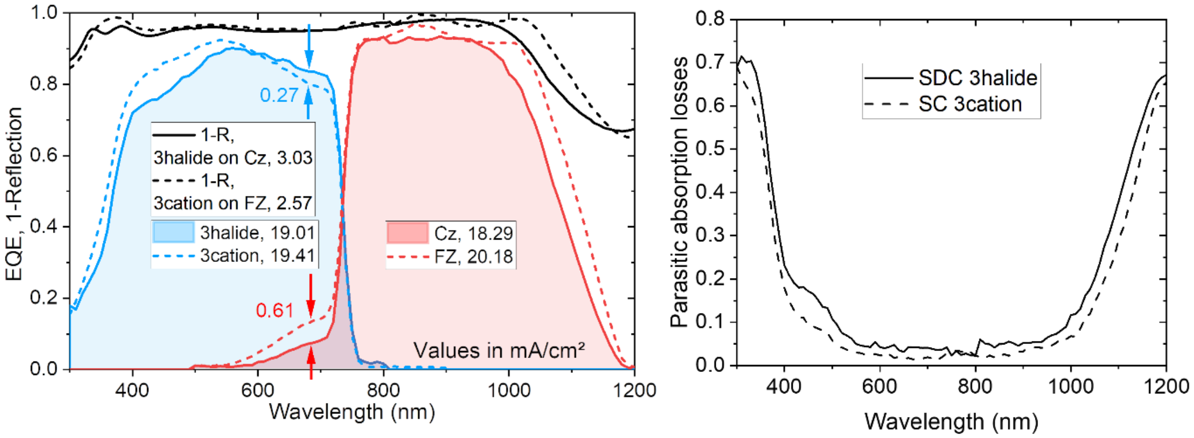

Figure S18. Left: EQE and 1-reflection (black line) of the champion tandem cell with SDC 3halide perovskites (23nm C_{60}) measured in-house. The spin coated triple cation (18 nm C_{60}) perovskite silicon tandem was used for direct comparison. The data is extracted from previous literature[6]. Right: the parasitic absorption loss over the spectrum between 300 nm to 1200 nm.

The investigation of the reproducibility issue of the slot-die coated perovskite silicon solar cell

It has been reported previously that a silver contact layer can degrade because halides from the perovskite layer could react with silver into silver halides [11]. Due to our coating process and the 5 µm back textured silicon, the perovskite solution could easily infiltrate into the silicon rear side during coating which could not be perfectly sealed. Therefore, the rear electrode could be easily eroded in the slot-die process. Although we have tried ethanol and EDTA to clean the degraded electrode and brushed additional silver paste, no conductive enhancement has been observed which meant the residue was still attached and indissoluble. The significant series resistance reduction leads to a direct FF improvement.

For a better understanding of this effect, we conducted the Transfer Length Method (TLM) to examine the sheet resistance and contact resistance between silver and rear side TCO underneath the silver. This data is displayed in Figure S20. After the extraction of the total resistance along various contact distances, the slope of the two linear relations can represent the sheet resistance of the TCO. This sheet resistance increases 16 times after being in contact with the perovskite solution. At the same time, the increased contact resistance has been increased from 1.81 Ohm to 8 Ohm. The contact resistance indicates that the silver surface has deteriorated into silver halides inducing for higher resistivity. To proof our assumption and prevent the undesired silver reaction, we sputtered an additional 25 nm of ITO onto the rear side to protect the silver back electrode. However, as seen in the SEM images in Figure S19, it showed that the ITO couldn't stop the penetration. In a second approach we used 25 nm SiO_x to protect the rear side of the silicon bottom cell. The approach was successful: 1 % diluted hydrofluoric acid (HF) has been used to remove this protection layer after the whole tandem solar cells was completed. The new device architecture is illustrated in Figure S21A. The performance deviation has been narrowed down from 12% to 4% after introducing this protective and sacrificial SiO_x layer on the back side. Within this batch, with confirmed current density by EQE in Figure S21B, the best performance cell's J-V scans are shown in Figure S22. Again, the silicon bottom cell limits the matched current to 17.8 mA/cm² due to a silicon wafer thickness of only 105 µm. When we implemented the variated spectral illumination, the best FF achieves 76.13 for the perovskite junction. According to the previous reported result [6], our slot-die coated perovskite silicon tandem solar cell using 2PACz as HTL has approached the medium performance and reached the same charge extraction as spin coated perovskite tandem cells.

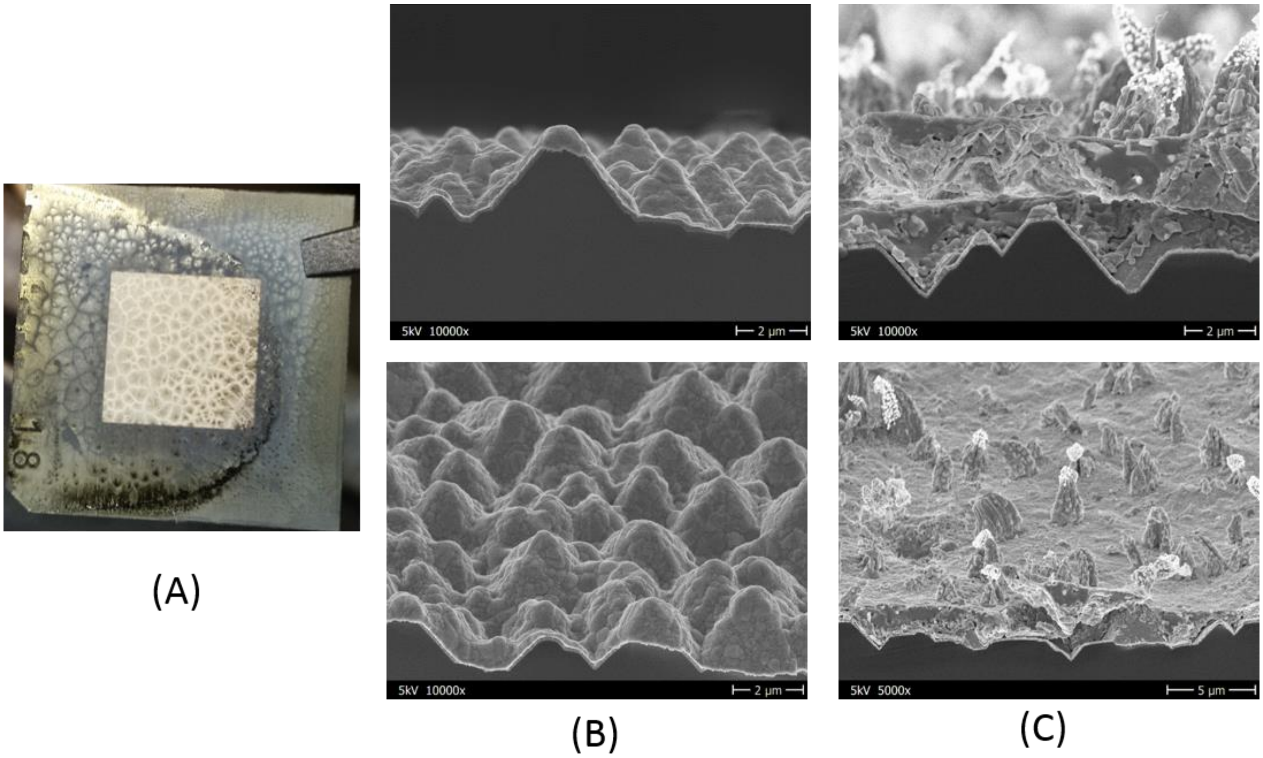

Figure S19. (A) The degraded silver back electrode after slot-die coating; (B) SEM image of the original silver electrode without reaction with perovskite solution; (C) SEM image of the silver electrode after reaction with perovskite solution and annealed on a hotplate. The potential silver halides are generated, such as AgI, AgBr, AgCl and PbO were generated [11].

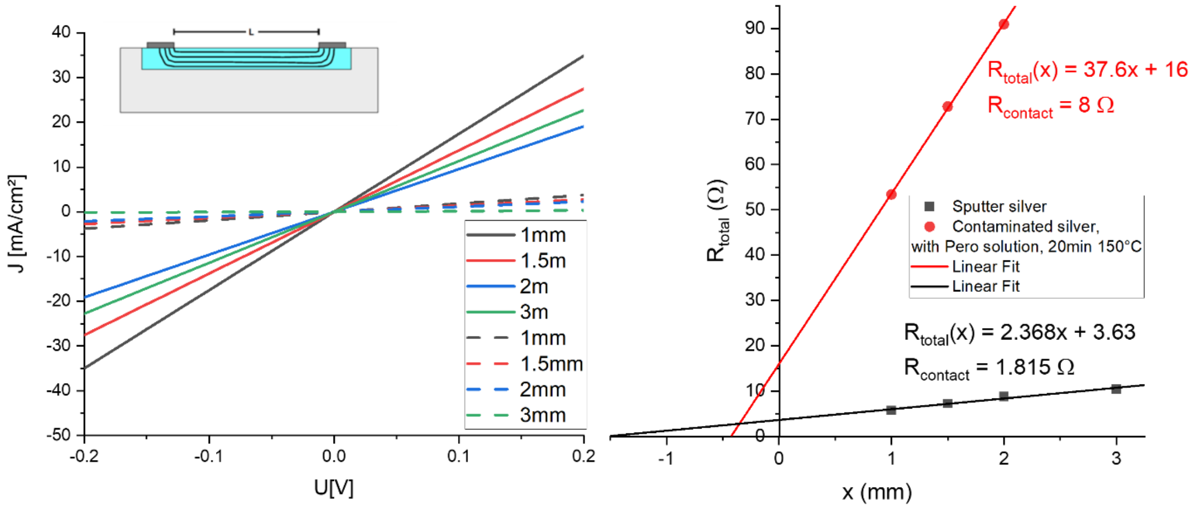

Figure S20. Left: The TLM sample was prepared by SiN between nSCOT and Cz wafer as insulating layer. Then 150 nm nSCOT and 400 nm Ag electrode was sputtered on wafer (Cz/SiN/nSCOT/Ag). The perovskite solution was dropped on the top directly. After removing extra solution by gravity, the silver

side was attached onto hotplate to simulate the standard annealing condition. The color lines were solid lines before halide perovskites erosion while the dashed lines were after halide perovskites erosion and 20 min annealing at 150°C. The distance from 1 mm to 3 mm corresponds to the Ag pads distance. Right: extraction of the data from TLM measurement. The regression indicates the contact resistance between silver and TCO increases from 1.815 Ohm to 8 Ohm. And the slope indicates the sheet resistance increases almost 16 times which may indicate the halide perovskites have also reacted with the TCO layer.

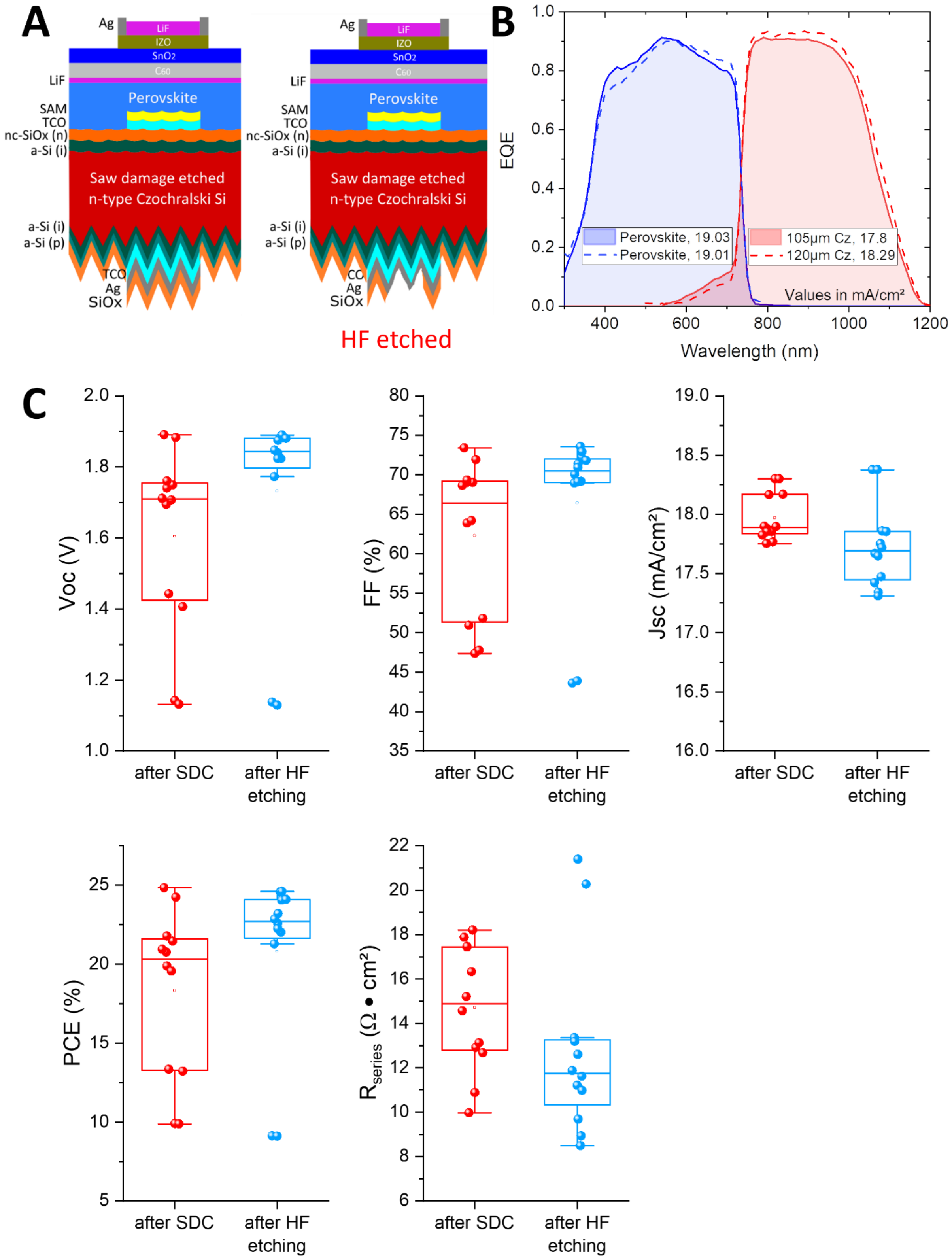
A
Ag
LiF
IZO
SnO2
C60
LiF
Perovskite
SAM
TCO
nc-SiOx (n)
a-Si (i)
Saw damage etched
n-type Czochralski Si
a-Si (i)
a-Si (p)
TCO
Ag
SiOx
HF etched
B
EQE
Perovskite, 19.03
Perovskite, 19.01
105µm Cz, 17.8
120µm Cz, 18.29
Values in mA/cm²
Wavelength (nm)
C
Voc (V)
FF (%)
Jsc (mA/cm²)
PCE (%)
Rseries (Ω • cm²)
after SDC
after HF etching

Figure S21. (A) Another 25 nm SiO_x has been added by PECVD to protect the back side. After the whole fabrication process, 1% HF solution has been used to remove the SiO_x protection layer. The 100 nm silver electrode has also been slightly damaged. In Figure S16, the silver paste has been brushed after erasing to achieve better contact. (B) The EQE comparison between 120 µm and 105 µm silicon wafer. The current density of perovskite layer was almost the same. However, the current mismatch is caused by the insufficient silicon absorption. (C) Here was a batch which has been measured before and after HF etching. The light intensity has been the same for both measurements. Afterwards, the light intensity has been calibrated as silicon limited performance with 17.8 mA/cm². The lower current generated by silicon was due to the reduced silicon thickness from 120 µm to 100 µm. The series resistance values are calculated at the slope of the V_{oc} intersect points.

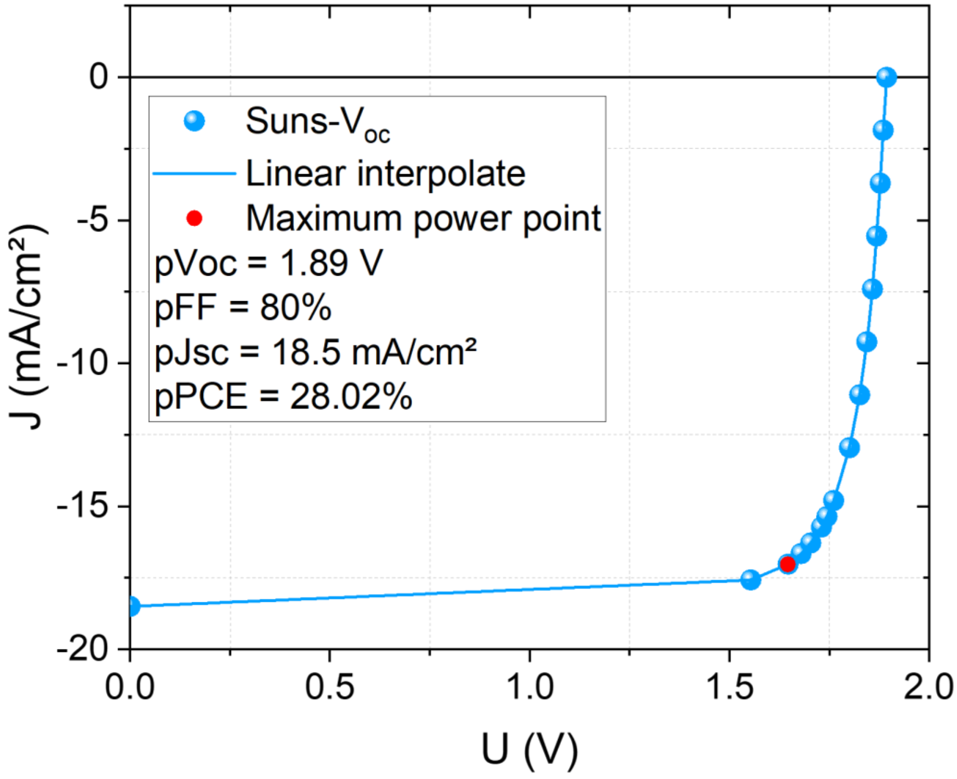

Figure S22. The obtained pseudo J-V curve of the tandem solar cell to quantify the implied efficiency potential in absence of any transport or serial resistance losses. The maximum power point is marked in red in panel.

References

1. Mazzarella, L., Morales-Vilches, A.B., Hendrichs, M., Kirner, S., Korte, L., Schlatmann, R., and Stannowski, B. (2018). Nanocrystalline n-type silicon oxide front contacts for silicon heterojunction solar cells: Photocurrent enhancement on planar and textured substrates. IEEE J. Photovoltaics *8*, 70–78.

2. Kegel, J., Angermann, H., Stürzebecher, U., Conrad, E., Mews, M., Korte, L., and Stegemann, B. (2014). Over 20% conversion efficiency on silicon heterojunction solar cells by IPA-free substrate texturization. Appl. Surf. Sci. *301*, 56–62.

3. Köhnen, E., Jošt, M., Morales-Vilches, A.B., Tockhorn, P., Al-Ashouri, A., Macco, B., Kegelmann, L., Korte, L., Rech, B., Schlatmann, R., et al. (2019). Highly efficient monolithic perovskite silicon tandem solar cells: Analyzing the influence of current mismatch on device performance. Sustain. Energy Fuels *3*, 1995–2005.

4. Stolterfoht, M., Grischek, M., Caprioglio, P., Wolff, C.M., Gutierrez-Partida, E., Peña-Camargo, F., Rothhardt, D., Zhang, S., Raoufi, M., Wolansky, J., et al. (2020). How To Quantify the Efficiency Potential of Neat Perovskite Films: Perovskite Semiconductors with an Implied Efficiency Exceeding 28%. Adv. Mater. *32*, 2000080.

5. Rabbani, A., and Ayatollahi, S. (2015). Comparing three image processing algorithms to estimate the grain-size distribution of porous rocks from binary 2D images and sensitivity analysis of the grain overlapping degree. Spec. Top. Rev. Porous Media *6*, 71–89.

6. Al-Ashouri, A., Köhnen, E., Li, B., Magomedov, A., Hempel, H., Caprioglio, P., Márquez, J.A., Morales Vilches, A.B., Kasparavicius, E., Smith, J.A., et al. (2020). Monolithic perovskite/silicon tandem solar cell with >29% efficiency by enhanced hole extraction. Science (80-.). *370*, 1300–1309.

7. Hou, Y., Aydin, E., De Bastiani, M., Xiao, C., Isikgor, F.H., Xue, D.J., Chen, B., Chen, H., Bahrami, B., Chowdhury, A.H., et al. (2020). Efficient tandem solar cells with solution-processed perovskite on textured crystalline silicon. Science (80-.). *367*, 1135–1140.

8. Xu, J., Boyd, C.C., Yu, Z.J., Palmstrom, A.F., Witter, D.J., Larson, B.W., France, R.M., Werner, J., Harvey, S.P., Wolf, E.J., et al. (2020). Triple-halide wide-band gap perovskites with suppressed phase segregation for efficient tandems. Science (80-.). *367*, 1097–1104.

9. Chen, B., Yu, Z.J., Manzoor, S., Wang, S., Weigand, W., Yu, Z., Yang, G., Ni, Z., Dai, X., Holman, Z.C., et al. (2020). Blade-Coated Perovskites on Textured Silicon for 26%-Efficient Monolithic Perovskite/Silicon Tandem Solar Cells. Joule *4*, 850–864.

10. Subbiah, A.S., Isikgor, F.H., Howells, C.T., De Bastiani, M., Liu, J., Aydin, E., Furlan, F., Allen, T.G., Xu, F., Zhumagali, S., et al. (2020). High-performance perovskite single-junction and textured perovskite/silicon tandem solar cells via slot-die-coating. ACS Energy Lett. *5*, 3034–3040.

11. Svanström, S., Jacobsson, T.J., Boschloo, G., Johansson, E.M.J., Rensmo, H., and Cappel, U.B. (2020). Degradation Mechanism of Silver Metal Deposited on Lead Halide Perovskites. ACS Appl. Mater. Interfaces *12*, 7212–7221.
